# Extending semiconductor plasmonics to the UV: Fano resonances and disorder engineering in Ag–In–S nanoparticles

**DOI:** 10.1039/d6tc02675c

**Published:** 2026-10-26

**Authors:** Biljana Pejova, Julio A. do Nascimento, Rola Moussa, Fayzah Talbi, Adam Kerrigan, Leonardo Lari, Richard Douthwaite, Ljupcho Pejov, Vlado K. Lazarov

**Affiliations:** a Institute of Chemistry, Faculty of Natural Sciences and Mathematics, SS. Cyril and Methodius University, POB 162 1000 Skopje North Macedonia biljana@pmf.ukim.mk; b School of Physics Engineering and Technology, University of York York YO10 5DD UK vlado.lazarov@york.ac.uk; c Department of Physical Sciences, Faculty of Science, University of Jeddah P.O. Box 13151 Jeddah 21493 Saudi Arabia; d The York-JEOL Nanocentre, University of York York YO10 5DD UK; e Area Science Park Padriciano 99 34149 Trieste Italy; f Department of Chemistry, University of York York YO10 5DD UK

## Abstract

We report widely tunable plasmonic resonance in ternary Ag–In–S and quaternary Ag–In–S–Zn semiconductor nanoparticles (NPs), that exhibit intraband free-carrier localized surface plasmon resonance (LSPR) in the NIR and interband-driven plasmon/plexcitonic Fano mode in the UV spectral regions (300–350 nm), extending semiconductor plasmonics to short wavelengths. These plasmonic properties are precisely tailored by controlling electronic structure, including energy band gaps and Urbach energies in addition to free charge carrier concentration *via* extrinsic Zn-doping and sonochemically controlled structural disorder. While NIR LSPR bands are symmetric, the UV plasmonic peaks are notably asymmetric and can be modelled with a Fano-like band shape function. The nature of the asymmetry is quantum-mechanical *i.e.* a Fano-like resonance between a continuous plasmon state and a discrete electronic excitation. By interpolating the experimental interband plasmonic bands in the UV region with a generalized Fano line shape function, relevant parameters such as plasmon damping, resonance energy and asymmetry factor were obtained. In addition, we show that a classical electrodynamics approach based on a driven-damped oscillator also reproduces the asymmetry. The demonstrated plasmon-exciton coupling in Ag–In–S based NPs, which is tunable *via* both external doping and sonochemical processing, offers a new platform for potential applications requiring precise control of plasmon absorption across a wide spectral region.

## Abstract 2

Ternary Ag–In–S and quaternary Ag–In–S–Zn semiconductor NPs exhibit intraband free-carrier LSPR in the NIR and interband-driven plasmon/plexcitonic Fano mode in the UV spectral region, extending semiconductor plasmonics to short wavelengths.

## Graphical abstract

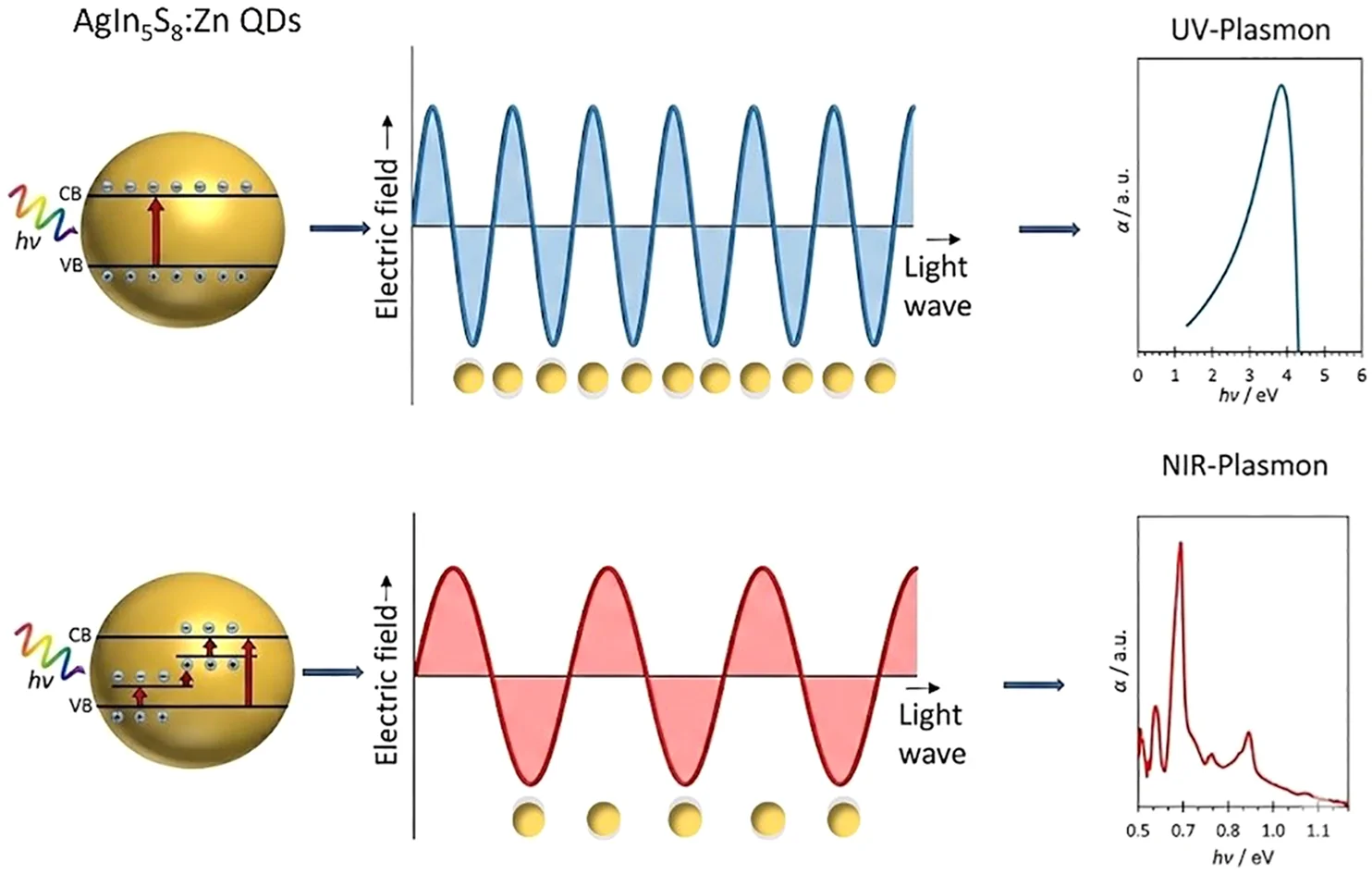

## 1. Introduction

One of the most effective strategies for tuning optoelectronic properties of semiconductor nanostructures is intentional insertion of foreign atoms/ions into their lattice *i.e.* doping.^1–8^ Along with quantum size effects, this strategy additionally modifies the band structure of bulk semiconductors enabling precise control of optical and electrical properties for tailored applications. Substitutional doping with heterovalent atoms/ions introduces impurity levels in the gap between valence and conduction bands that consequently leads to filling the conduction band (case of n-doping) with free charge carriers *i.e.* electrons or holes (case of p-doping) resulting in a Burstein–Moss-shift of the optical band gap energy.^1^ Free charge carriers are responsible for electron/hole transport processes, as well as modification of both bulk and surface plasmons. The collective oscillation of confined charge carriers in response to incident radiation can lead to the phenomenon of localized surface plasmon resonance (LSPR). Therefore, heterovalent doping in semiconductors opens many possibilities for new applications and technologies governed by plasmonic effects such as sensors, photodetectors, waveguides, smart windows, biosensors, bio-imaging, photothermal therapy, energy harvesting and photocatalysis.^1,3,5^

Conventional metal-based plasmonic materials, such as the noble metals Au and Ag, have electron density of the order of 10^22^–10^23^ cm^−3^ which is almost fixed and cannot be modulated with post-synthetical treatments. In contrast, the charge carrier density of semiconductor nanostructures is several orders of magnitude lower (10^18^–10^21^ cm^−3^) and can be efficiently controlled by the dopant concentration. This provides the opportunity for a tunable plasmonic response over a broad spectral range spanning from visible and near-IR to the THz domain.^1–9^ In particular, doped NPs, prepared using a bottom-up colloidal approach, are amenable to synthetic modification and post-synthetic treatments. However, the ability to reliably control the doping and consequently the free charge-carrier concentration in NPs remains a challenge and it is essential to their functionality as systems with modulable electronic and optical properties.

Here we demonstrate the use of an aliovalent doping mechanism to tailor the free charge carrier concentration in colloidally prepared AgIn_5_S_8_ NPs and consequently their LSPR and interband plasmon bands. Moreover, we demonstrate experimental synthesis conditions significantly influence the level of structural order of synthesized AgIn_5_S_8_ NPs, which provides an additional pathway to tailor plasmonic properties of AgIn_5_S_8_ NPs without doping. Besides the expected plasmonic resonances in the NIR region, AgIn_5_S_8_ NPs have additional asymmetric plasmonic bands in the near UV-UV (300–350 nm), observed for the first time in these systems, providing an opportunity for plasmon tuning over a large spectral range from NIR to UV. We also show that in the UV-region the plasmon resonance peak exhibits asymmetry, which is the result of a resonant coupling between plasmons and excitons. This phenomenon can be understood semiclassically, as well as quantum mechanically by using the Fano resonance concept which allows determination of the damping/coupling parameters between the interacting continuum and discrete states. Applications governed by resonant plasmon-exciton coupling have been described, which has further stimulated the search for plasmonic semiconductor nanomaterials that allow pathways for tuning of LSPR and ultimately achieving high-energy plasmon absorption.^10^ Our results on UV plasmons of AgIn_5_S_8_ NPs here confirm literature assumptions for unique plasmonic properties of ternary semiconductor nanocrystals dependent on the cation composition/distribution.^11^ For example, G. Gabka *et al.* reported an extraordinarily large composition-induced tunability of LSPR for ternary Cu–Fe–S nanocrystals.^12^ The LSPR absorption maximum shifts from ∼490 nm for Cu/Fe = 1 to ∼1200 nm for Cu/Fe = 6.22. Similarly, Liu *et al.* have shown that ternary antimony-doped Sb_*y*_–Cu_*x*_S nanocrystals have tunable plasmonic resonances from near-infrared to the mid-visible spectral region by adjusting the Sb content.^13^ The work presented here shows that ternary NPs such as AgIn_5_S_8_ can exhibit tunable plasmon resonances beyond the visible region, offering a new platform for semiconductor plasmonic applications, with a high potential for control of the degree of chemical and structural order and ultimately optical properties.

## 2. Experimental

### 2.1. Synthesis of ternary Ag–In–S and quaternary Ag–In–Zn–S nanostructures

For the purpose of our study, we have synthesized four types of nanostructures: undoped AgIn_5_S_8_ NPs and extrinsically Zn doped NPs, AgIn_5_S_8_:Zn using ultrasound (US)-assisted route (AgIn_5_S_8_ NPs-US and AgIn_5_S_8_:Zn-US) as well as undoped and doped NPs obtained without US field (AgIn_5_S_8_ NPs and AgIn_5_S_8_:Zn NPs). All syntheses were performed by a modified experimental procedure developed by our group based on the bottom-up colloidal approach.^14^ Silver nitrate, AgNO_3_, indium(iii) sulfate, In_2_(SO_4_)_3_, zinc nitrate, Zn(NO_3_)_2_ and thioacetamide, (CH_3_)CS(NH_2_), were used as precursors of relevant metal cations (Ag^+^, In^3+^, Zn^2+^) and S^2−^ anions respectively. Sodium dodecyl sulfate (SDS), C_12_H_25_NaO_4_S and sodium thiosulfate, Na_2_S_2_O_3_, were included in the reaction system too. SDS had the role of a surfactant, while Na_2_S_2_O_3_ as a ligand agent controlled and retained low concentration of the metal cations providing suitable conditions for formation of AgIn_5_S_8_ lattice.

#### 2.1.1. Synthesis of AgIn_5_S_8_:Zn and AgIn_5_S_8_:Zn-US NPs

The developed experimental procedure for these two nanostructures includes preparation of two solutions in separate containers. The chemical composition of the first solution is 4.0 × 10^−2^ mol L^−1^ In^3+^, 1.0 × 10^−2^ mol L^−1^ Ag^+^, 3.0 × 10^−1^ mol L^−1^ S_2_O_3_^2−^, 3.0 × 10^−1^ mol L^−1^ (CH_3_)CS(NH_2_) and 3.5 × 10^−4^ mol L^−1^ C_12_H_25_NaO_4_S, while the second one contains 3.0 × 10^−2^ Zn^2+^ mol L^−1^, 2.0 × 10^−1^ mol L^−1^ Na_2_S_2_O_3_ and 2.0 × 10^−1^ mol L^−1^ (CH_3_)CS(NH_2_). Both solutions were heated from room temperature to 75 °C and maintained at this temperature shortly until their transformation to yellow and white turbid suspensions respectively. The complex balanced equilibria, taking place in both solutions, lead to formation of AgIn_5_S_8_ NPs and ZnS NPs respectively. In the second step of the developed procedure, 50 cm^3^ of both solutions were mixed and maintained at 75 °C for 90 min to give AgIn_5_S_8_:Zn or sonicated for 60 min at 75 °C to give AgIn_5_S_8_:Zn-US. The sonication was done by immersion of an ultrasound horn, using a home-made setup which generates ultrasound waves with frequency of 20 kHz and intensity of 100 W cm^−2^. The growth of doped AgIn_5_S_8_:Zn NPs-US occurred under multi-bubble sonoluminescence (MBSL) conditions for 60 min before cooling to room temperature. The obtained samples were separated using filtration, washed with doubly deionized water and dried at room temperature.

#### 2.1.2. Synthesis of undoped AgIn_5_S_8_ NPs

The AgIn_5_S_8_ NPs were prepared under chemical and sonochemical conditions using the first reaction solution from the previously described experimental procedure. The NPs growth took place at 75 °C for 60 min in case of sonochemical route, while when the reaction system was not treated by ultrasound irradiation the temperature of 75 °C was maintained for 90 min before allowing to cool down to room temperature. The prepared samples were separated using filtration, washed with doubly deionized water and dried at room temperature.

### 2.2. Experimental methods

The properties of synthesized nanostructures were characterized using scanning electron microscopy (SEM), transmission electron microscopy (TEM), powder X-Ray diffraction (PXRD), UV-VIS, NIR and Raman spectroscopy. SEM images were measured on a JEOL 7800F Prime SEM. TEM selected area electron diffraction (SAED) patterns, as well as high resolution TEM (HRTEM) images were obtained using a JEOL 2100+ TEM and aberration corrected JEOL-NeoARM STEM/TEM. Diffraction patterns were simulated using JEMS software, while the TEM-SAED patterns were calibrated using Au nanoparticles as a reference. The atomic interplanar spacings were measured from selected areas of HRTEM images, using fast-Fourier transform (FFT). Measurements of PXRD patterns of the synthesized NPs were carried out on ultima IV X-ray diffractometer (Rigaku Co.) in the *θ*/2*θ* mode (step-scan of Δ(2*θ*) = 0.02°) using Cu Kα radiation. UV-VIS spectra were measured in the spectral range from 200 nm to 1100 nm on a Cary 50 spectrophotometer (HP Co.) in transmission mode and presented as normalized absorption. NIR spectra were measured in the spectral range from 0.5 eV to 1.25 eV on ANTARIS FT NIR spectrometer.

## 3. Results and discussions

### 3.1. Structure and composition of AgIn_5_S_8_ and AgIn_5_S_8_:Zn NPs

Fig. 1, shows representative atomic resolution images from the 4 different AgIn_5_S_8_ synthesized NPs, comprising undoped and doped materials, obtained with and without ultrasound (US). The NPs have a spherical-like morphology with no pronounced surface faceting and some of the atomic planes characteristic for the spinel phase of AgIn_5_S_8_ are labeled in Fig. 1.

**Fig. 1:**
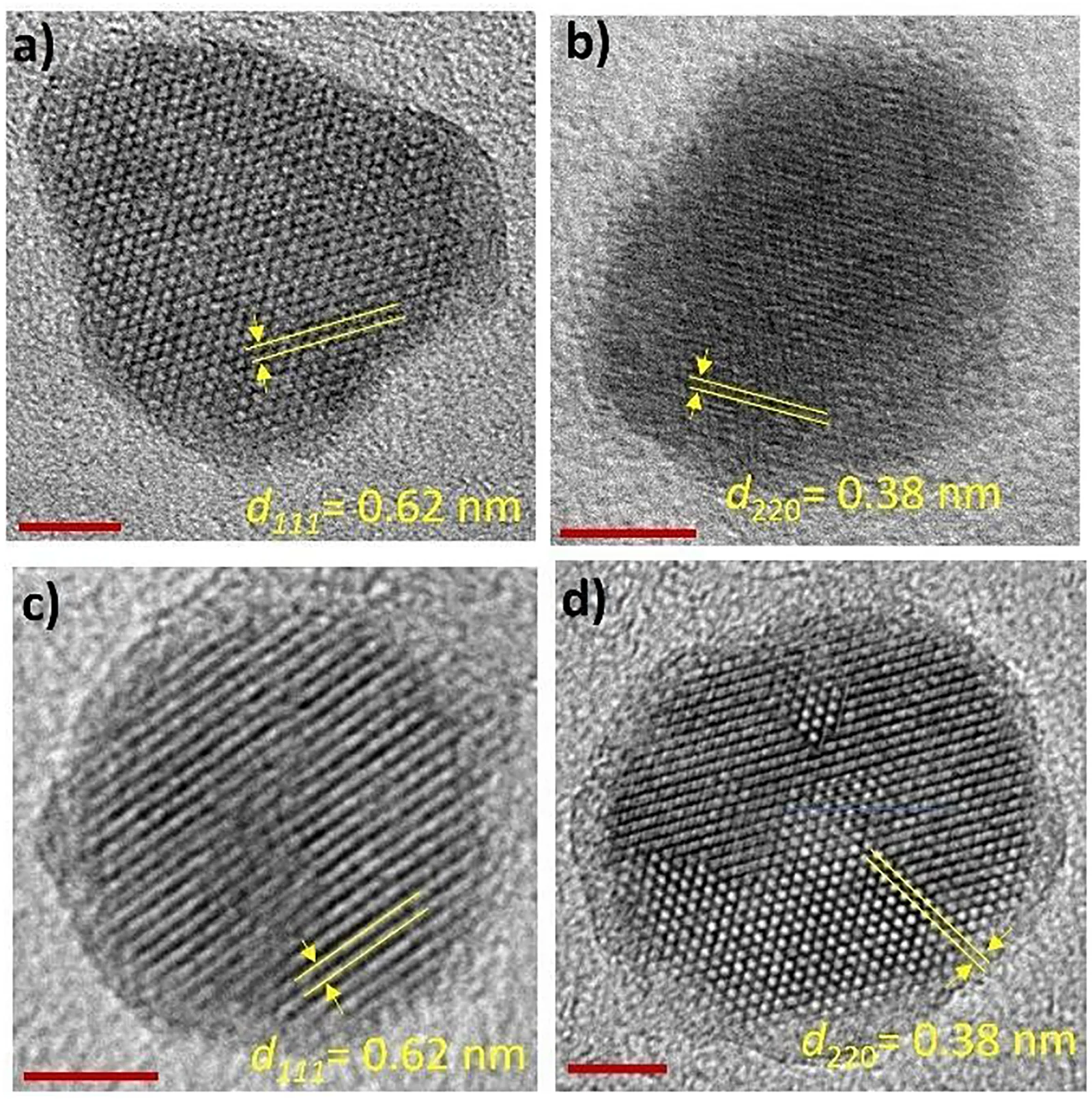
HRTEM images of the NPs: (a) AgIn_5_S_8_, (b) AgIn_5_S_8_-US, (c) AgIn_5_S_8_:Zn and (d) AgIn_5_S_8_:Zn-US. Representative lattice *d*-spacings are outlined for each NP. All scale bars are set to be 5 nm.

Transmission electron microscopy (TEM) particle size measurements of AgIn_5_S_8_:Zn and AgIn_5_S_8_:Zn-US gave 14.5 ± 6.0 nm and 21.4 ± 6.4 nm for the average NP diameters, respectively. The average diameter of undoped AgIn_5_S_8_ is 5.8 ± 2.2 nm while undoped AgIn_5_S_8_-US shows a bimodal distribution of 4.5 ± 1.1 nm and 15.9 ± 4.1 nm. We note that use of US results in NPs with larger diameter.

Furthermore, US irradiation increases the crystallinity of AgIn_5_S_8_ NPs, as evidenced by the selected areas electron diffraction (SAED) and PXRD, (Fig. 2 and SI-Fig. S1) and subsequently influences the optical properties (see below).

**Fig. 2:**
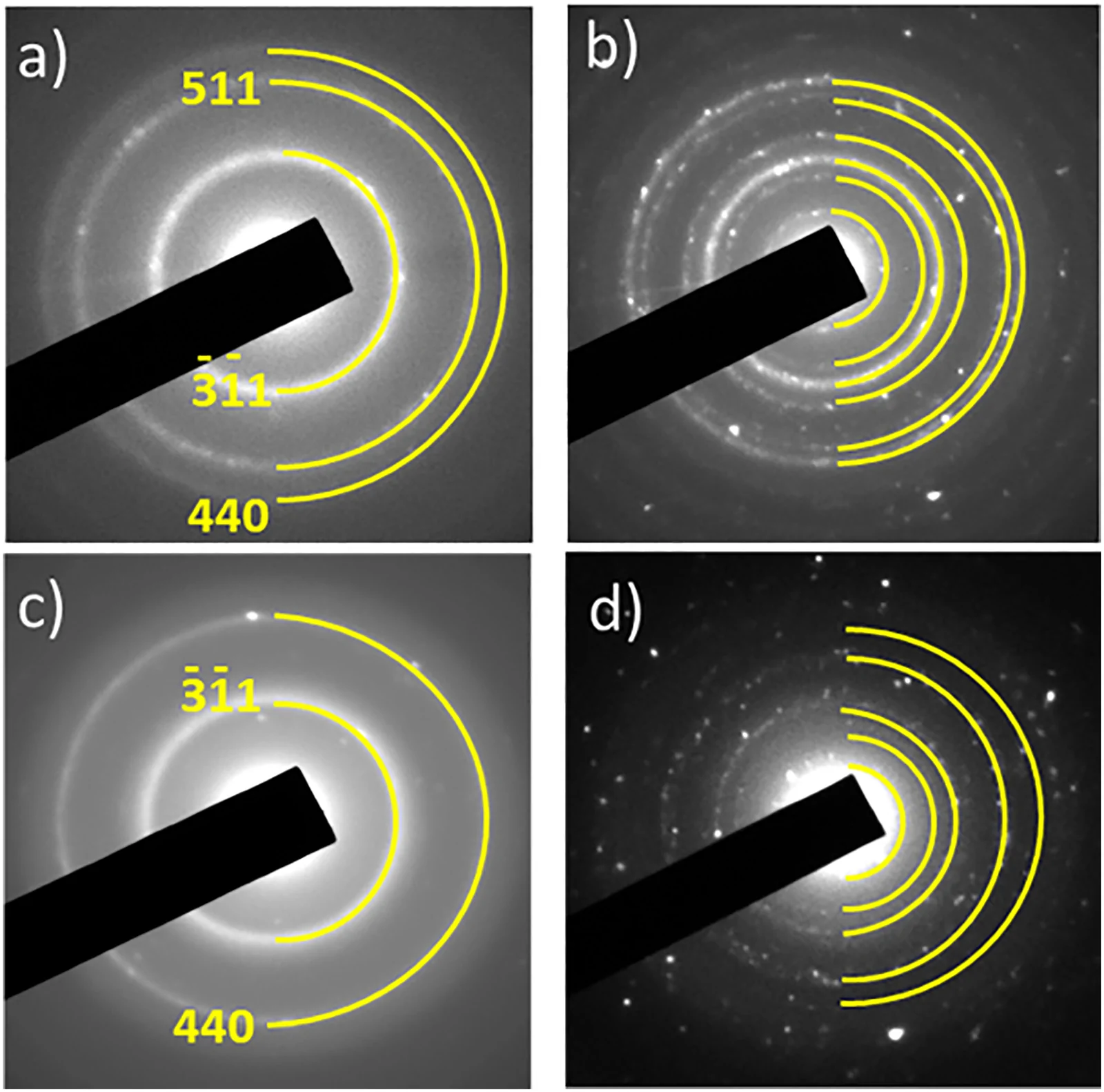
Selected area electron diffraction of the studied NPs: (a) AgIn_5_S_8_, (b) AgIn_5_S_8_-US, (c) AgIn_5_S_8_:Zn and (d) AgIn_5_S_8_:Zn-US annotated with their corresponding Miller indices. In (b) and (d) the rings, going outwards, correspond to (111), (220), (311), (400), (511) and (440).^15^

Energy-dispersive X-ray spectroscopy (EDS) of AgIn_5_S_8_:Zn and AgIn_5_S_8_:Zn-US shows that US facilitates the doping process, leading to a slightly higher Zn atomic ratio of 9.4% for AgIn_5_S_8_:Zn-US compared to 6.8 at% for AgIn_5_S_8_:Zn and mapping shows that Zn^2+^ ions are homogeneously distributed (Fig. 3). The dopant distribution of Zn^2+^ within the NP can have effects on the optical properties, as shown by S. D. Lounis *et al.* when dopant segregates and leads to a core shell formation NPs.^16^

**Fig. 3:**
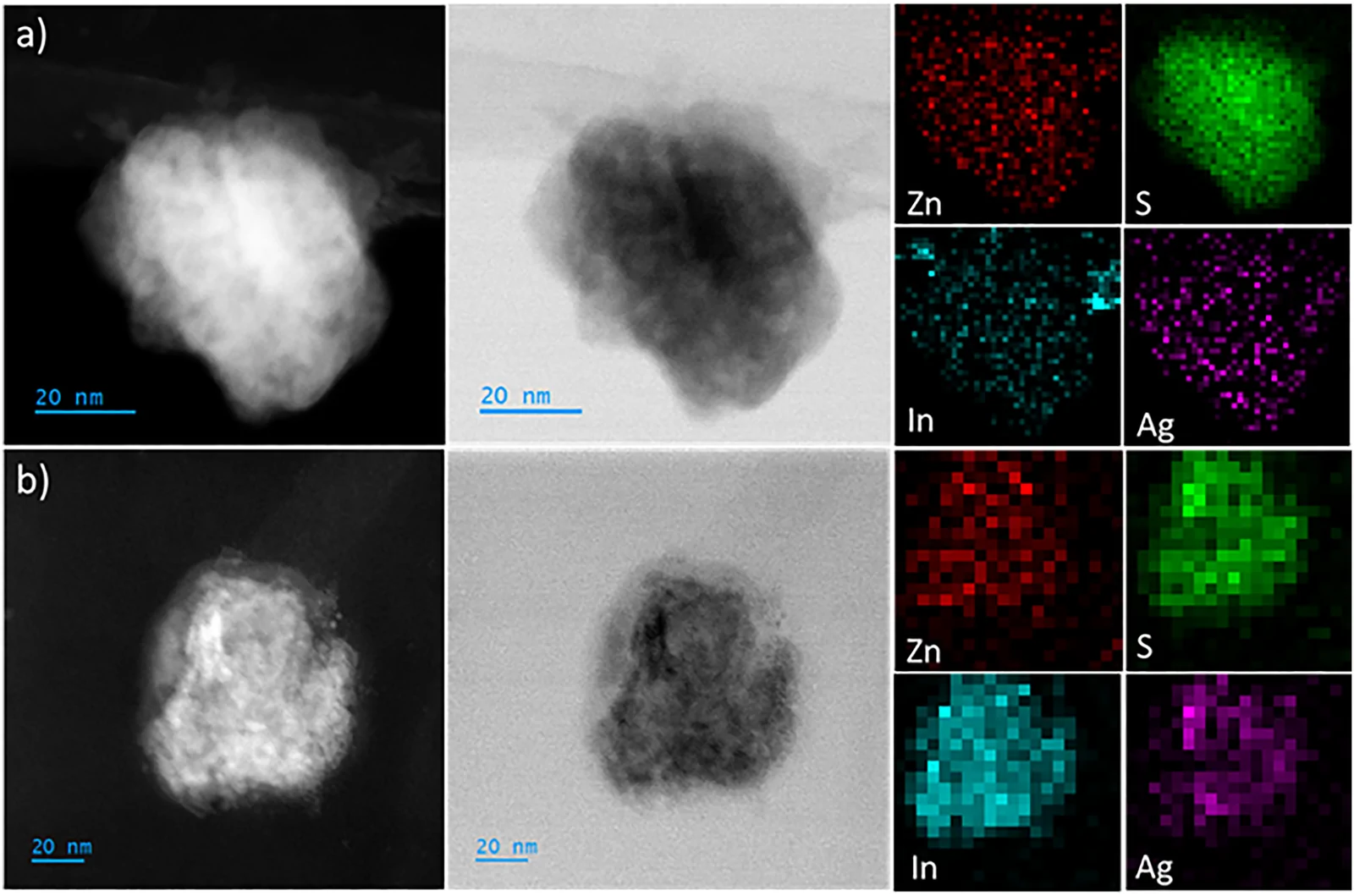
Images and elements maps for the studied NPs: (a) AgIn_5_S_8_:Zn-US and (b) AgIn_5_S_8_:Zn.

SEM-EDX measurements (Table 1) suggest that Zn^2+^ is incorporated into the AgIn_5_S_8_ lattice *via* extrinsic aliovalent doping by substituting predominantly In^3+^. This is supported by similar ionic radii of tetrahedrally coordinated Zn^2+^ (*r*(Zn^2+^) = 74 pm) and In^3+^ (*r*(In^3+^) = 76 pm in tetrahedral *vs.* 94 nm in octahedral coordination geometry) compared to Ag^+^ (*r*(Ag^+^) = 126 pm).^17^ Also, the most common coordination number of Zn^2+^ is 4, leading to the most probable scenario for incorporation of Zn^2+^ into the AgIn_5_S_8_ lattice through substitution of host In^3+^ or Ag^+^ at the tetrahedral sites. The occupation of cation sites in AgIn_5_S_8_ single bulk-crystals is 80% In^3+^ on octahedral sites and 20% of In^3+^ and Ag^+^ ions on tetrahedral sites corresponding to the general formula (AgIn)In_4_S_8_ (AB_2_X_4_).^18^

**Table 1:** Chemical composition of synthesized NPs obtained by SEM-EDX analysis

Atomic ratio/%	AgIn_5_S_8_	AgIn_5_S_8_:Zn	AgIn_5_S_8_-US	AgIn_5_S_8_:Zn-US
S	59.9 ± 2.6	59.6 ± 2.3	60.9 ± 3.6	61.5 ± 1.6
Zn	—	6.8 ± 0.8	—	9.4 ± 0.5
Ag	9.7 ± 0.6	9.3 ± 0.4	9.3 ± 0.7	6.8 ± 0.4
In	30.4 ± 2.0	24.3 ± 1.9	29.8 ± 2.8	22.3 ± 0.8

### 3.2. Band gap and Urbach energy changes due to doping and US

UV-vis spectroscopy of AgIn_5_S_8_ and AgIn_5_S_8_-US allows determination of their band gap energies of 2.45 ± 0.03 and 2.66 ± 0.06 eV, respectively, which are blue shifted compared to bulk AgIn_5_S_8_ reported between 1.7–1.9 eV.^19–22^ The blue shift is characteristic of a size confinement effect where NPs have a radius smaller than the Bohr exciton radius. Indeed, AgIn_5_S_8_ has a greater particle size (5.8 ± 2.2 nm) than AgIn_5_S_8_-US (4.5 ± 1.1 nm) while the band gap energy is smaller.

However, even though doped AgIn_5_S_8_:Zn and AgIn_5_S_8_:Zn-US are on average ∼2–3 times larger in comparison to undoped AgIn_5_S_8_, their band gap energies are greater (Fig. 4a and b) at 2.88 ± 0.02 eV and 2.96 ± 0.02 eV, respectively indicating that doping dominates in these systems.^1–8^ Doping of AgIn_5_S_8_ with Zn^2+^ alters the band structure by introducing impurity levels in the gap between the valence and conduction bands. These states contribute free carriers in the valence or conduction band, which leads to increase of *E*_g_, as a consequence of the Burstein–Moss effect. The slightly higher value of *E*_g_, found for AgIn_5_S_8_:Zn-US, correlates with the larger dopant ratio compared to AgIn_5_S_8_:Zn obtained by SEM-EDX (Table 1).

**Fig. 4:**
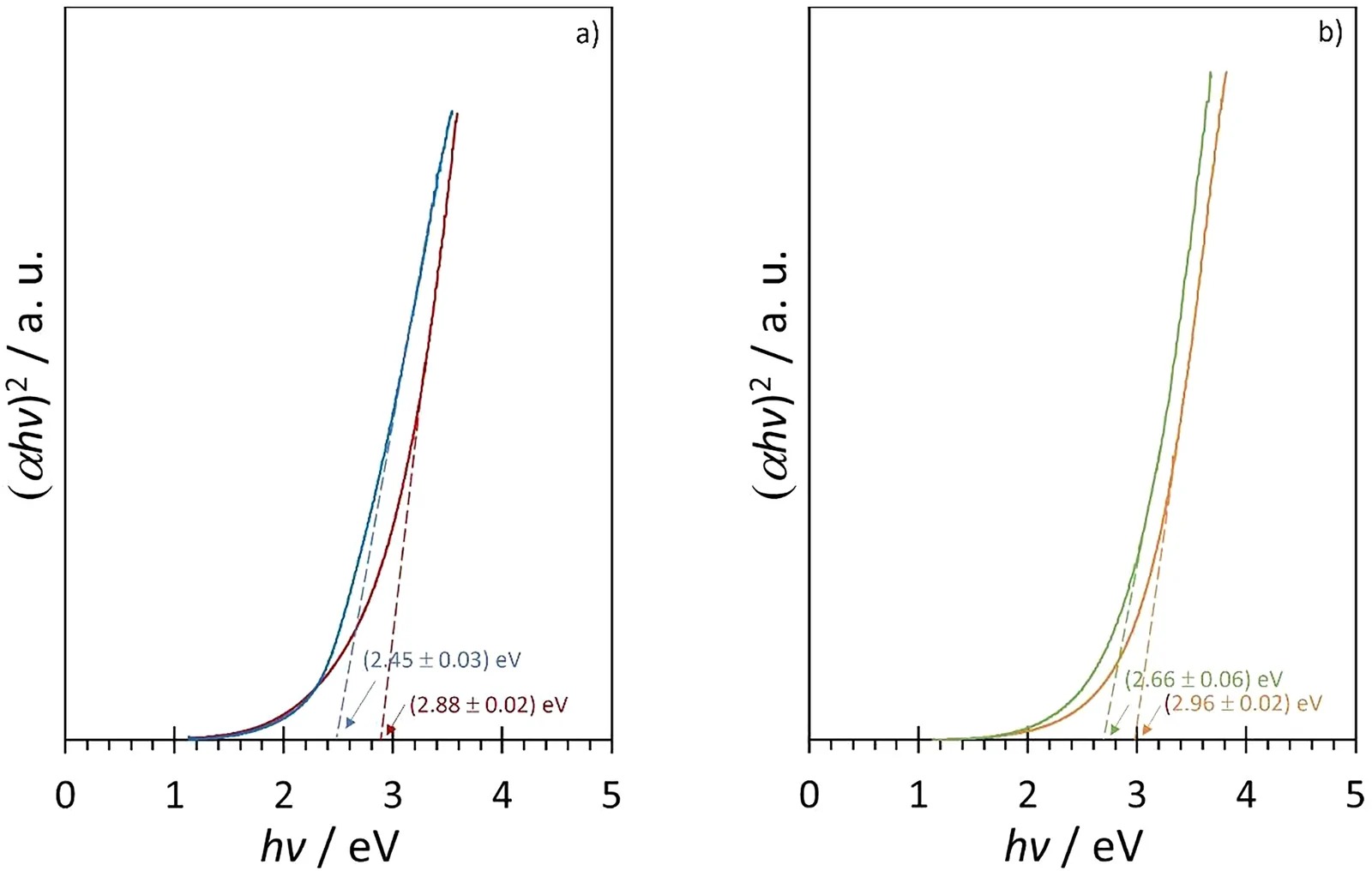
(*αhv*)^2^ on *hv* dependences of synthesized NPs: (a) AgIn_5_S_8_ (blue) and AgIn_5_S_8_:Zn (red), and (b) AgIn_5_S_8_ US (green) and AgIn_5_S_8_:Zn-US (orange).

Urbach energies (*E*_u_) from the sub-band gap absorption data were used to estimate the degree of structural disorder and its evolution with respect to doping and US (Table 2). The Urbach energy of AgIn_5_S_8_:Zn-US is larger than AgIn_5_S_8_-US, 1049 ± 17 meV *vs.* 258 ± 96 meV, whereas in case of AgIn_5_S_8_:Zn and AgIn_5_S_8_ are 1537 ± 92 meV and 916 ± 22 meV, respectively indicating that Zn^2+^ ions increase the structural disorder and US increases crystallinity as also shown by SAED (Fig. 2).

**Table 2:** Band gap (*E*_g_) and Urbach (*E*_U_) energies of synthesized NPs obtained by UV-Vis spectroscopy

	AgIn_5_S_8_ QDs	AgIn_5_S_8_:Zn QDs	AgIn_5_S_8_ QDs-US	AgIn_5_S_8_:Zn QDs-US
*E*_g_/eV	2.45 ± 0.03	2.88 ± 0.02	2.66 ± 0.06	2.96 ± 0.02
*E*_U_/meV	916 ± 22	1537 ± 92	258 ± 96	1049 ± 17

The selected area electron diffraction in Fig. 2b and d confirms above discussion *i.e.* that the NPs synthesized using US approach show better crystallinity than the corresponding NPs obtained without sonication. The electron diffraction from US prepared NPs (Fig. 2b) show less diffusive diffraction rings and more distinct diffraction spots which indicates improved crystallinity which ultimately results with the smallest range of the Urbach energy, 258 ± 96 meV in the synthesized NPs.

### 3.3. Plasmonic absorption of zinc-doped and undoped NPs in the UV-VIS and NIR parts

Normalized absorption spectra of AgIn_5_S_8_, AgIn_5_S_8_-US, AgIn_5_S_8_:Zn, and AgIn_5_S_8_:Zn-US (Fig. 5a and b) show intense absorption peaks in the UV-Vis region with *λ*_max_ ∼ 330 nm. On the basis of in-depth analysis of our experimental data, we attribute these transitions to interband plasmons, additionally modified as a consequence of quantum interference between exciton and plasmon states, leading to Fano line shapes, as we show further. We first show the interband plasmon character of these transitions. Optical transitions in a given material are encoded in the complex dielectric function:^23^

1 *ε*(*ω*) = *ε*_1_(*ω*) + *iε*_2_(*ω*)

where the real (*ε*_1_(*ω*)) and imaginary (*ε*_2_(*ω*)) part of *ε*(*ω*) are related by the Kramers–Kronig transformation. In case of a plasmonic platform based on non-interacting free charge carriers, which experience only instantaneous collisions with the nuclei (such as, *e.g.* noble metal plasmonics), optical response is determined by the Drude model for *ε*(*ω*):2
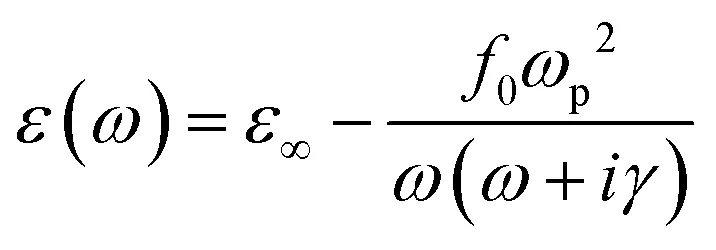


**Fig. 5:**
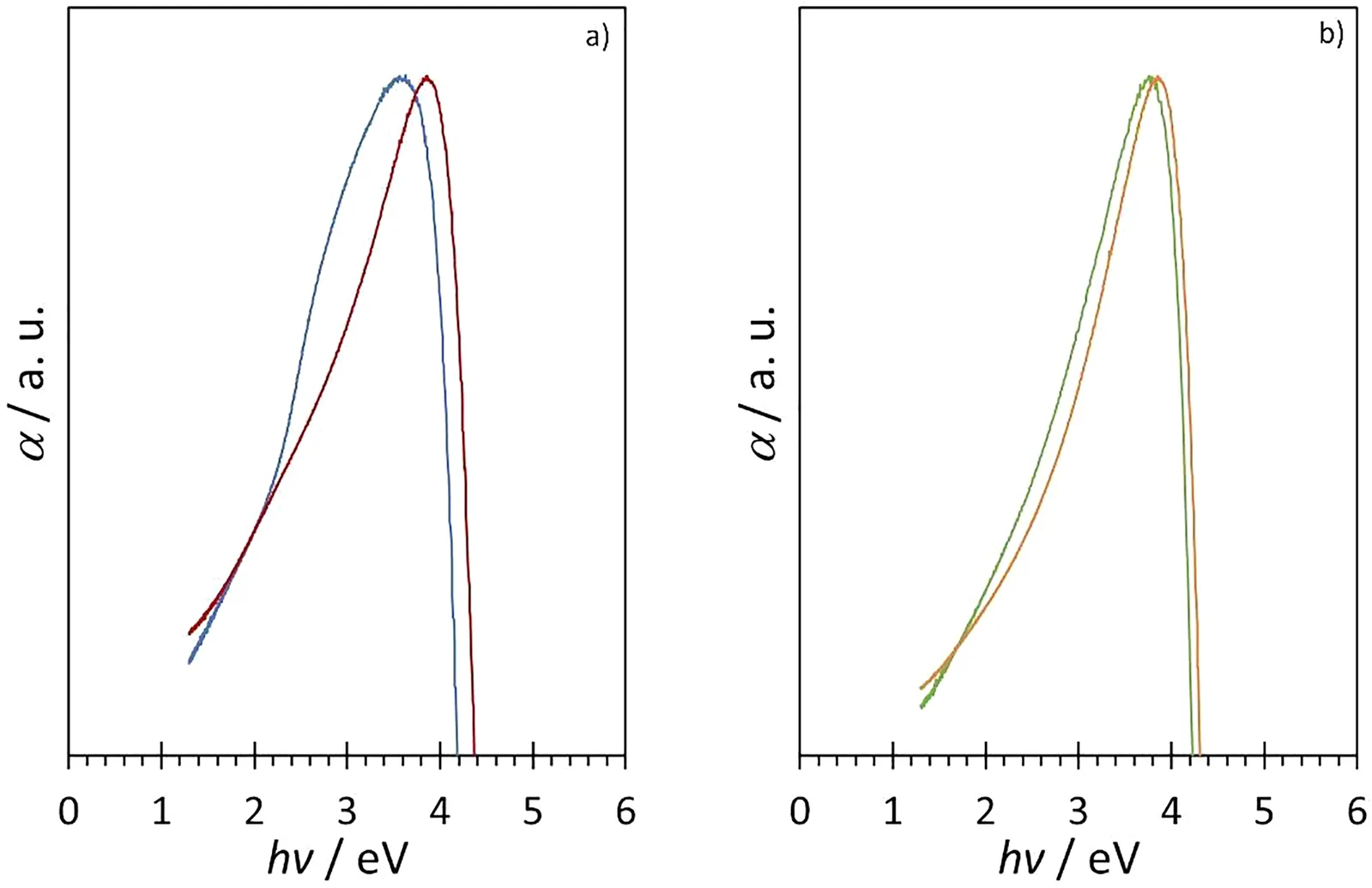
Normalised absorption spectra of the synthesized NPs: (a) AgIn_5_S_8_ (blue) and AgIn_5_S_8_:Zn (red) and (b) AgIn_5_S_8_-US (green) and AgIn_5_S_8_:Zn-US (orange).

In (2), *ε*_∞_ is the high-frequency dielectric constant of the material, *ω*_p_ its plasma frequency, *f*_0_ is the free-electron oscillator strength, while *γ* is the damping constant related to the collision frequency. Deducing the explicit forms of *ε*_1_(*ω*) and *ε*_2_(*ω*) from (2), it can be shown that the resonance condition is fulfilled when *ε*_1_(*ω*) < 0.

In case of intrinsically or extrinsically doped semiconductor plasmonic materials, strong absorption in the energy range surpassing the band gap value leads to generation of charge carriers in the conduction band, which determines both components of the complex dielectric function. In such case, the Drude model should be augmented by Lorentz terms, which account for the relevant interband contributions:^23^3
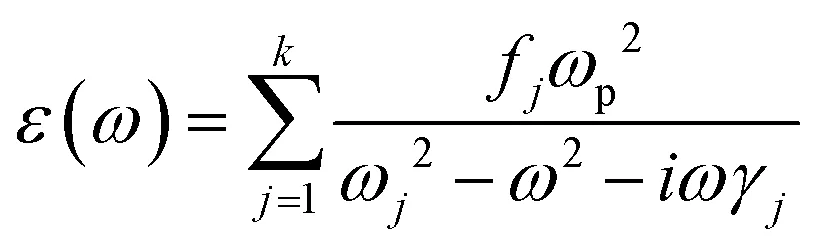
where *f*_*j*_ is the oscillator strength of the *j*-th transition, while *ω*_*j*_ and *γ*_*j*_ are the corresponding frequency and damping factor. Strong absorption in the vicinity of the band gap, corresponding to high values of *ε*_2_(*ω*) produce anomalous dispersion in *ε*_1_(*ω*), which can lead to negative values at photon energies above the band gap, even when the contribution from free charge carriers is insufficient. As demonstrated by Gjerding *et al.*^24^ for layered van der Waals crystals and further elaborated by Darabian *et al.*^25^ in their compendium of natural epsilon-near-zero (ENZ) materials, interband electronic transitions with sufficiently high oscillator strength can produce negative *ε*_1_(*ω*) in the UV-Vis range even in the complete absence of Drude free-electron contribution. This mechanism has been demonstrated experimentally in semiconductors such as amorphous silicon (α-Si), where the material exhibits *ε*_1_(*ω*) < 0 in the UV to near-UV range (∼94–317 nm) entirely due to interband contributions. In the presently studied system, AgIn_5_S_8_ with a cubic spinel structure (space group *Fd*3̄*m*), there are several high-symmetry critical points in the first Brillouin zone where the gradient of the interband energy difference vanishes (∇_*k*_[*E*_c_(*k*) − *E*_v_(*k*)] = 0) – Van Hove singularities.^26^ At these critical points the joint density of states either diverges or undergoes abrupt change in slope, which can produce pronounced interband transition features.^22,26^ The valence band maximum of AgIn_5_S_8_ is reported to have mixed Ag 4d and S 3p character, while the conduction band minimum is dominated by In 5s states.^18^ Such p–d hybridized valence states combined with s-like conduction states favor sizable transition matrix elements |*P*_*if*_(*k*)|^2^ at the Γ point and at the zone boundary X and L points, which can contribute to the relatively high oscillator strength of the interband transitions. Furthermore, when the material is synthesized in nanocrystalline form, quantum confinement constrains the oscillator strength into a few discrete low-energy transitions, so that the lowest-energy allowed states carry a large fraction of the total transition strength per unit volume.^27^

To provide further proof of the outlined assignment, we have carried out a detailed analysis of the optical response of the AgIn_5_S_8_ and AgIn_5_S_8_:Zn NPs using dispersion models based on the complex dielectric function. The spectral response was parametrized using Drude–Lorentz model (DL) as well as an extended Drude–Lorentz model incorporating the critical point (CP) contributions (DL + CP).^28–30^ While the Drude model (without Lorentz and CP contributions) does not reproduce the optical response of the studied samples, the DL as well as the DL + CP models provide excellent fits of the experimental data. We note that fitting was successful even by a pure Lorentz-oscillator model. This finding, along with the obtained negative value of the *ε*_1_(*ω*) component in the relevant spectral range, further support the interband plasmon character of the absorption feature in the UV region. The obtained real and imaginary components of the complex dielectric function are provided in SI-Fig. S2 and S3. In the case of undoped AgIn_5_S_8_ NPs (SI-Fig. S2), it can be seen that while *ε*_1_(*ω*) is positive from 1100 to 337 nm, in the UV spectral range (starting at 337 nm, *i.e.* 3.68 eV), it attains negative values. The Fröhlich resonance condition Re[*ε*(*ω*)] = −2*ε*_m_(*ω*) is satisfied at *ħω* = 3.72 eV (*λ* = 333 nm) in the UV region, close to the observed band maximum. The interband plasmonic regime thus operates in the UV region *via* interband/high-frequency electron dynamics, as reported by Darabian *et al.*^25^ and Todorov *et al.*^31^ and is completely distinct from the visible interband transition onset. Instead of being due to free charge carriers, the negative value of *ε*_1_(*ω*) is in this case a consequence of semiconductor's electronic bands acting as elastically bound resonators. Similar behavior is exhibited by the doped AgIn_5_S_8_:Zn NPs (SI-Fig. S3). Unlike conventional localized surface plasmon resonances (LSPR) governed by Drude-like intraband free carriers, the resonance observed here corresponds to an interband plasmon. In the subwavelength dipolar limit, the electrodynamics resonance condition still formally derives from the Fröhlich criterion. However, because *ε*(*ω*) in this spectral region is dictated by interband transitions, strong field dispersion and elevated damping cause the maximum in the optical response to deviate from the exact zero-crossing of Re[*ε*(*ω*)] + 2*ε*_m_ = 0.

The relative contributions of scattering and absorption to the optical response of AgIn_5_S_8_ and AgIn_5_S_8_:Zn NPs were evaluated using a dielectric function-based electromagnetic model (see SI for details), and these calculations confirmed that the absorption dominates the overall optical response (SI-Fig. S4 and S5). Therefore, the photoexcited electrons support interband plasmonic behavior of all 4 different sets NPs in this spectral range.

Notably *λ*_max_ of the interband plasmonic transitions correlates with the changes observed for band gaps which also blue shift on doping with Zn and use of US (Table 2). Due to the doping effect, the interband plasmonic peaks of these NPs are blue-shifted in comparison to undoped NPs.^1–8^ The small difference of dopant ratio in the doped NPs (∼6.8% Zn and ∼9.4% Zn) and small difference in their band gap energies of 0.08 eV leads to close positions (∼3.9 eV) of corresponding interband plasmonic peaks. In contrast, the corresponding peaks of undoped NPs (US) and undoped NPs, appear at separate energies, ∼3.8 eV and ∼3.6 eV, again reflecting the difference of 0.21 eV in their band gap energies. Of note, all interband plasmonic peaks in Fig. 5 exhibit asymmetry around the maximum with a steeper change in absorption coefficient at higher energy.

The asymmetry of plasmonic peaks, as well as their width will be discussed in more detail below. Here we focus on the quality factor *Q*, commonly used as a figure of merit for plasmons. *Q* is defined as the ratio between photon energy which corresponds to plasmonic peak maximum and the peak width, or alternatively through the components of the complex dielectric function:4
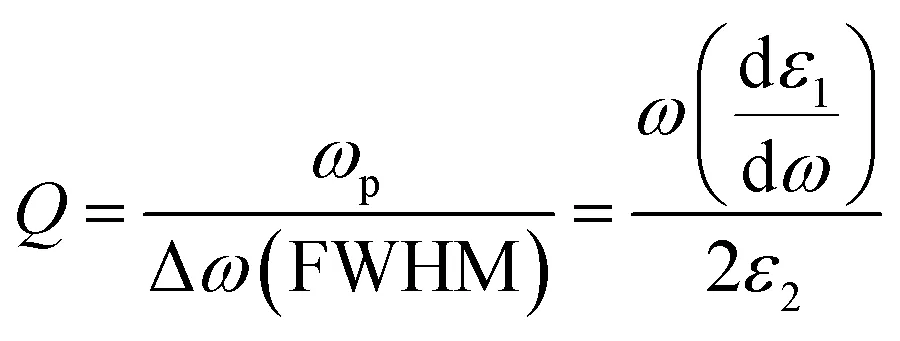


The relevant parameters for each plasmonic peak, its position and line width, were obtained by nonlinear least-squares fitting procedure with Fano-type function, discussed in details below. Interband plasmonic peaks of AgIn_5_S_8_:Zn-US and AgIn_5_S_8_-US, are characterized by a quality factor of 3.0 and 2.7, respectively. Similarly, AgIn_5_S_8_:Zn (*Q* = 2.4) has a larger *Q* than AgIn_5_S_8_ (*Q* = 2.1). The quality factors of these peaks correlate well with the structural/electronic properties discussed earlier which depend strongly on the presence of doping and US during synthesis. The increase in crystallinity using US is reflected in the *Q* factor increasing 25% and 28% for both doped and undoped NPs, respectively, while the doping itself increases the *Q* factor by 14% for NPs without US. Such *Q* values are lower compared to those typical for LSPR systems (which are often higher than 10). This is consistent with the interband plasmonic character of the reported resonances in our case. As demonstrated by Blaber *et al.*,^32^ when an electronic excitation coincides with interband transitions, the interband damping leads to substantial increase of *ε*_2_(*ω*) which, in turn, degrades the plasmon quality factor. The physical reason for this is the collective coherence being rapidly dephased *via* single-particle electron–hole pair generation (Landau damping), which causes underdamped collective oscillations in the relevant spectral window. Besides, the currently studied Ag–In–S and quaternary Ag–In–S:Zn NPs are characterized by a certain level of intrinsic structural and cationic disorder. The Ag^+^ and In^3+^ species tend to randomly share cation sublattices, inducing a cation disorder, while random incorporation of Zn^2+^ ions in the doped NPs creates significant potential fluctuations. Also, the significant concentration of native point defects such as cation vacancies (V_Ag_), anti-sites and interstitials generates high density of strong carrier scattering centers. The carrier relaxation rate in such disordered NPs is exceptionally high and induces ultrafast dephasing of any collective oscillation, causing significant broadening and damping of the coherent plasmonic behavior. All the previously outlined physical reasons (including strong interband absorption, electronic damping, structural and compositional disorder), along with the distribution of local dielectric environments, size dispersion of nanocrystals, and the Fano coupling to the continuum (discussed further), contribute to the apparent broad spectral features in the UV spectral region.

Besides the plasmonic response in the UV-Vis region, Fig. 6 shows additional plasmonic bands in the NIR region for all NPs. We attribute their appearance to the charge carriers injected into the conduction band either from the band gap states arising from the Zn-doping and/or lattice defects in NPs including the surface states. As for interband plasmons in the UV-Vis region, the NIR plasmon intensity and energy positions are modified on doping and using US during synthesis. AgIn_5_S_8_, exhibits peaks from 0.50–0.74 eV and a very intense plasmonic band at ∼1.2 eV. The appearance of this plasmonic band correlates with the increased charge carrier concentration with a higher level of structural disorder (*i.e.* less crystallinity), as well as higher Urbach energies. The improved structural order by sonication significantly decreases the intensity of the dominant ∼1.2 eV peak in AgIn_5_S_8_-US. Fig. 6b shows that the peak at ∼1.2 eV is almost absent for AgIn_5_S_8_:Zn-US, with plasmonic peaks occurring at ∼0.5, 0.7 and 0.9 eV. We also note that the plasmonic peaks in the NIR region are symmetric in contrast to the asymmetric plasmonic peaks in the UV-Vis region. To estimate the charge carrier concentrations in the studied materials, we have relied on detailed Raman band shape analysis of the longitudinal optical phonon–plasmon coupled modes (LOPC),^33–37^ which has been shown to lead to a correct order of magnitude as compared to the values obtained by other techniques. The charge carrier concentrations determined by this method were shown to vary within the range from ∼2.5 × 10^18^ to ∼2.5 × 10^20^ cm^−3^ for the samples with highest dopant content. Such concentrations correspond to plasma frequencies in the observed NIR region (from 0.13 to 1.17 eV), in excellent agreement with our experimental observations.

**Fig. 6:**
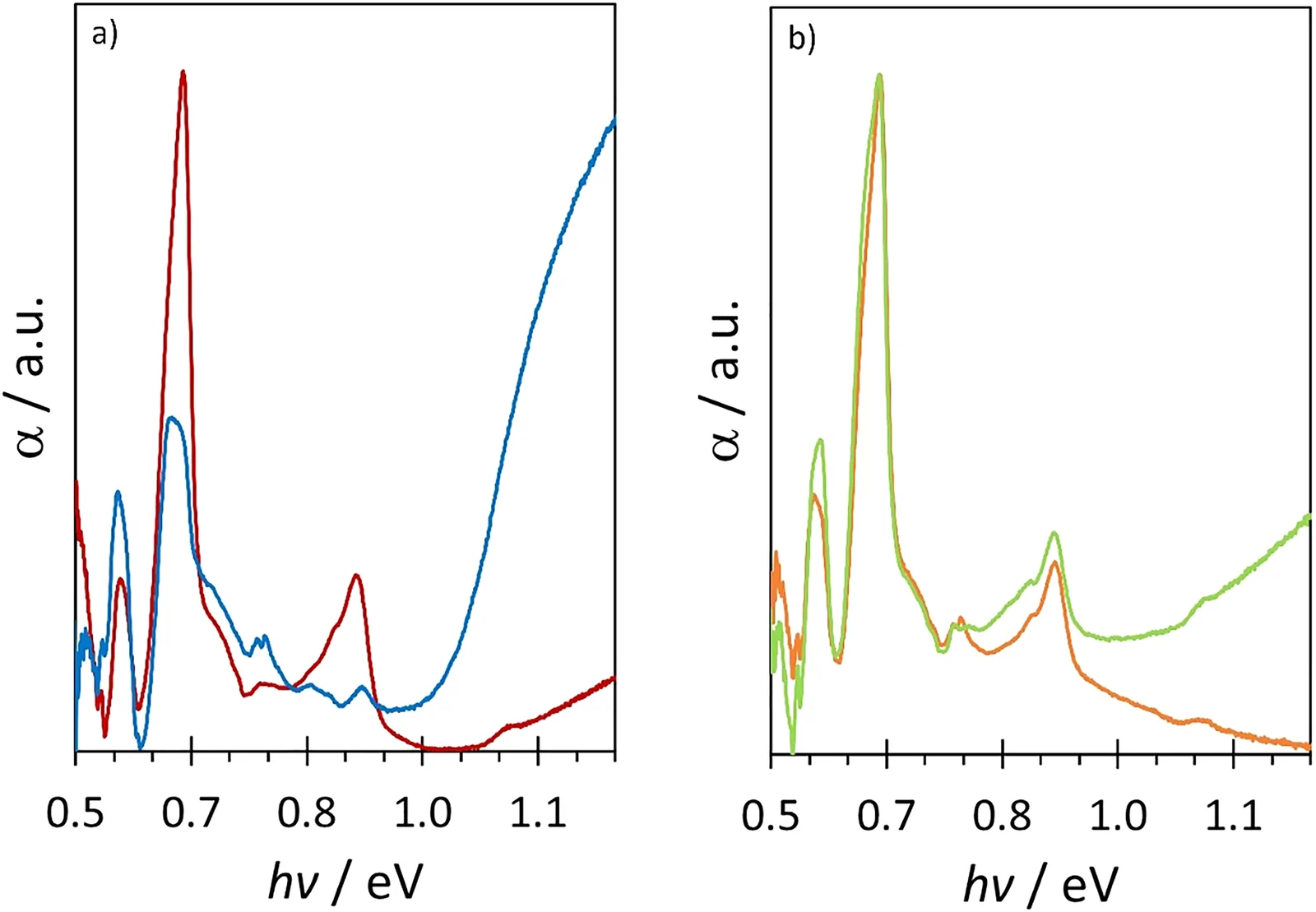
NIR plasmonic peaks of NPs showing the effect of the sonication on (a) AgIn_5_S_8_ (blue) and AgIn_5_S_8_-US (red) and (b) AgIn_5_S_8_:Zn (green) and AgIn_5_S_8_:Zn-US (orange).

Above results show good correlation between structural ordering, preparation methods *e.g.* presence/absence of US field as well as doping on the main features present in the plasmonic bands both in NIR and UV-vis region. In the next section we provide detailed modeling that explains the origin of asymmetric peaks which is closely correlated with the mechanisms of the plasmon decays.

#### 3.3.1. Modelling the plasmonic response of AgIn_5_S_8_-derived NPs

The experimental data show that the plasmonic behavior of AgIn_5_S_8_-derived NPs is complex. As already noted, interband plasmonic bands in the UV-Vis spectral region (Fig. 5) are characterized with pronounced asymmetry and we show here that they can be fitted with Fano-type functions. This indicates involvement of Fano-type resonance in the observed plasmonic phenomena.^38–43^ In contrast, the NIR plasmonic bands (Fig. 6), are more symmetrical and can be fitted with Cauchy–Lorentzian functions. To provide deeper understanding of the observed plasmonic properties, explain the notably asymmetric plasmonic absorption line-shape functions in the UV-Vis region, and to assign exact physical significance to the fitting parameters, we elaborate classical and quantum models.

From a classical modelling perspective, we consider two specific cases of driven-damped oscillator systems: linear and non-linear. To provide a quantum-level understanding of the NPs plasmonic properties, we combine a parameter-free modelling approach with a hybridization model for plasmon resonance phenomena, initially developed for complex metallic nanostructures.^44–51^

An essential condition for a semiconducting (or dielectric) material to exhibit LSPR optical properties is a negative value of the real part of its dielectric function (*ε*_r_ < 0). The minimum carrier concentration (*N*_min_) for this condition to be fulfilled is, within the Drude–Lorentz model and the approximation of dipolar modes, expressed as:5
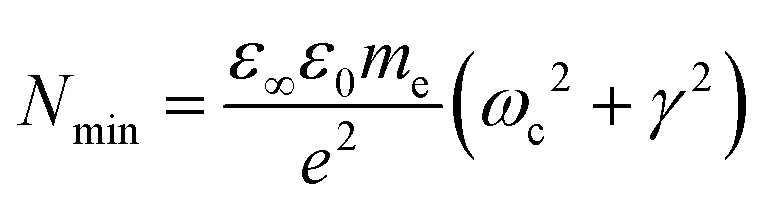
where *ε*_∞_ is the high-frequency dielectric constant of the material, *ω*_c_ is the cross-over frequency, *γ* is the damping of carrier oscillations (which includes ohmic and scattering losses), while all other symbols have their usual meaning. The bulk plasma frequency *ω*_p_ and the corresponding LSPR frequency *ω*_LSPR_ are both proportional to the square root of the charge carrier concentration:6
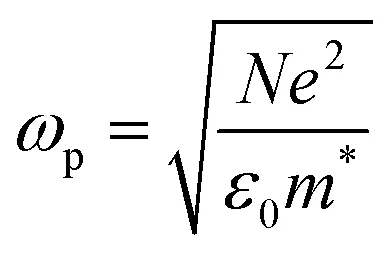
7
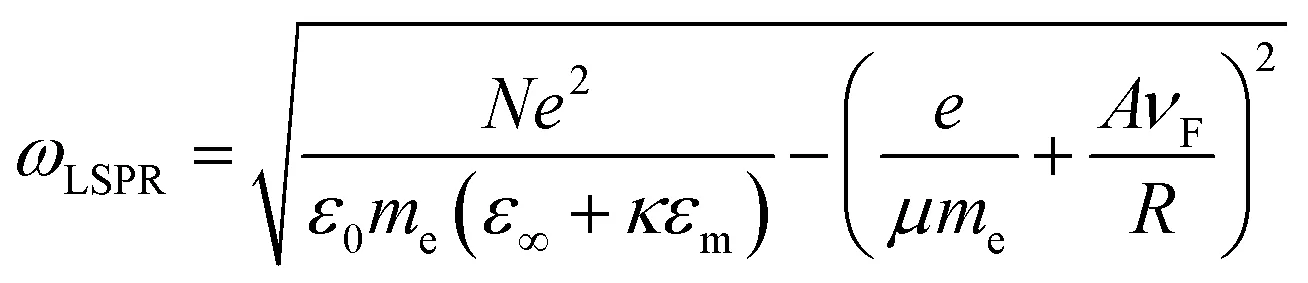


In eqn (7), *κ* is the surface geometric polarizability, *A* is a phenomenological parameter that can take values from the interval (0, 1), *μ* is the charge carriers’ mobility, *ν*_F_ is their Fermi velocity, *ε*_m_ the dielectric constant of the surrounding medium. In contrast to traditional plasmonic metals, doped semiconductors have lower carrier densities and consequently the LSPR responses are red shifted down to IR and even THz spectral regions. LSPR properties are dominantly governed by *N*, where *N* can be tailored within a range of several orders of magnitude in semiconducting nanostructures. Additional important factors that determine the LSPR frequency are *κ* and *ε*_m_, see eqn (7). While *κ* can be tuned by engineering of the NPs morphology, *ε*_m_ can be adjusted by modifying the medium which surrounds the NPs. The damping of the charge carrier oscillations, expressed through the parameter *γ* in (5), can be regarded as a superposition of several contributions, including electron–electron scattering, electron–phonon and electron-impurity scattering, inter and intra-band transition as well as surface-scattering.

#### 3.3.2. Classical oscillator models

The LSPR peaks observed in the NIR spectral region, can be attributed to the charge carriers injected into the conduction band from the band gap states arising either from extrinsic Zn-doping and/or lattice defects in NPs. Appearance of these plasmonic bands at much lower frequencies than those in the UV-Vis region is a consequence of much lower concentrations of free charge carriers (eqn (6) and (7)). In the case of AgIn_5_S_8_, where structural disorder is greater a very intense plasmonic band occurs at ∼1.2 eV in addition to peaks at 0.5–0.74 eV common to all NPs studied here (Fig. 6a). The appearance of multiple NIR-LSPR bands can be explained using the concept of plasmon hybridization in complex aggregates of semiconducting NPs, and all plasmonic peaks in the NIR spectral region can be fitted successfully with a Cauchy–Lorentzian lineshape function of the form:8
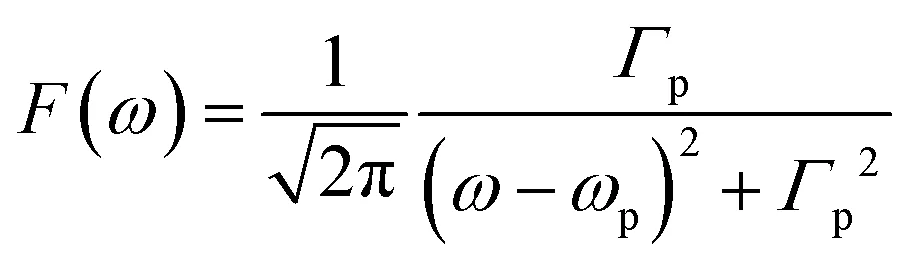


The Cauchy–Lorentzian distribution function is a Fourier transformation of the trajectory of an underdamped harmonic oscillator driven by the periodical electric component of incident light:

9 *x*(*t*) = e^−*Γ*_p_·*t*^ *C* cos(*ωt* + *φ*),

where *Γ*_p_ is plasmon damping parameter, *φ* is the phase shift, while *ω*_p_ is the resonance frequency of the plasmon. Therefore, physical interpretations may be assigned to the parameters of lineshape function (eqn (8)). The values of *Γ*_p_ and *ω*_p_ obtained by least-squares fitting to Cauchy–Lorentzian function of the form (eqn (8)) are summarized in SI-Table S1. The dephasing times, *τ*,10
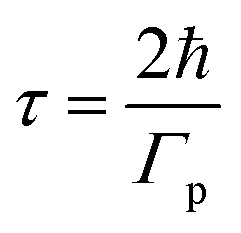
of the detected plasmonic modes calculated from the corresponding damping parameters were also calculated (SI-Table S1). Physically, the *τ* parameter represents the time period of decay of the plasmon amplitude. The calculated dephasing times fall within the range typical for plasmonics, from ∼10 to ∼50 fs.

To provide in-depth analysis of the interband plasmonic bands in the UV-Vis spectral region, and their asymmetric line Fano-like shapes, both classical and quantum mechanical formalisms may be invoked.

In the classical picture, we use two framework models. The first describes, the plasmonic oscillation represented by a driven-damped harmonic oscillator, with an equation of motion of the general form:11
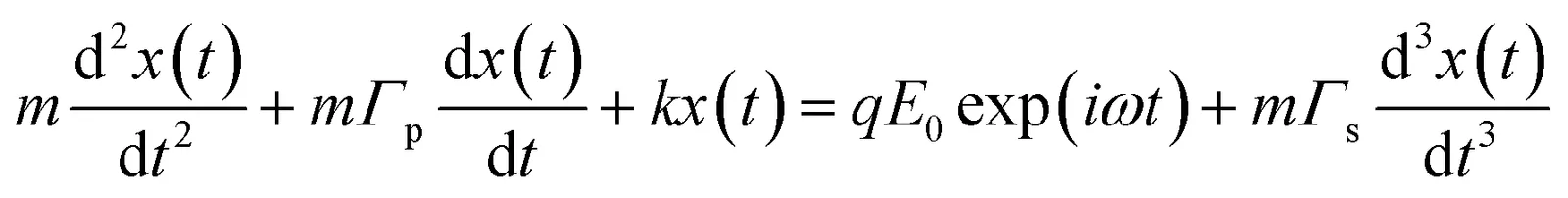


In eqn (11), aside from the nonradiative damping *Γ*_p_, a term due to the radiation damping (*Γ*_s_) is present, which is related to the recoil of the charge density related to radiation of photons, also known as Abraham–Lorentz reaction force. Setting *k* = 0 would eliminate the restoring force and apply to the case of free electrons, but here we use (11) to model the interband plasmonic response in the UV spectral range. The steady-state solution of (eqn (11)) has the form:12
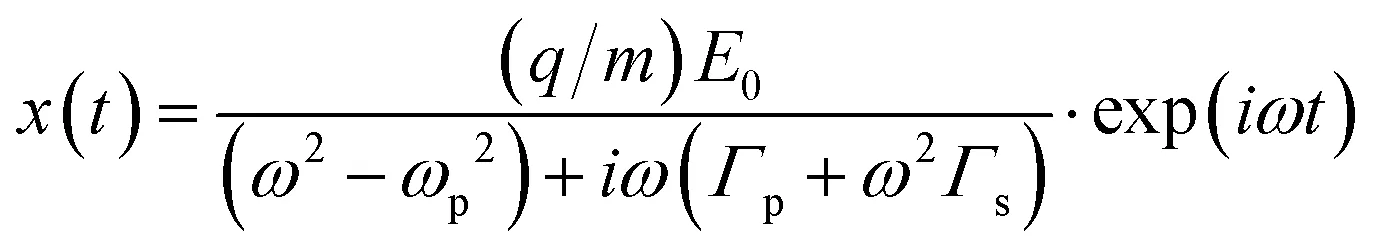


The absorption power spectrum is, correspondingly, given by:13
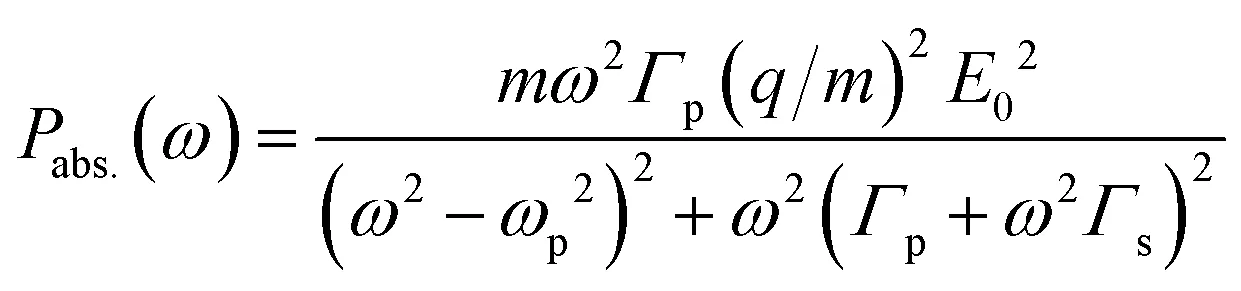
(see Fig. S6 in the SI for a typical fit of experimental data with a function of the form (eqn (13)). The *Γ*_p_ term can be split into the intra- and interband contributions:

14 *Γ*_p_ = *Γ*_p,intra_ + *Γ*_p,inter_

Intraband damping contribution, which is of central importance here, can be further represented as:15
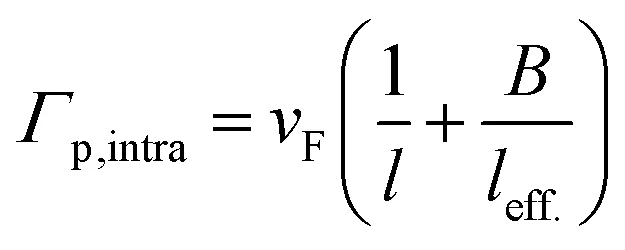


The first term in (eqn (15)) represents the electron–electron and electron–phonon interactions, and it is inversely proportional to the electron mean free path *l* and directly proportional to the Fermi velocity *v*_F_. The second term represents the electron–defect and electron–surface interactions and is known as the Landau or Kreibig damping mechanism. It becomes significant at small sizes of plasmonic nanoparticles, close or less than *l*, as well as in the case of high-density crystal defects, leading to *l*_eff._ < *l*.

Alternative to the above approach, a variant of the classical model can be applied, in which the plasmonic oscillation is treated as a nonlinear driven-damped oscillator. We hereby include a quartic anharmonic potential, *i.e.* the equation of motion becomes:16
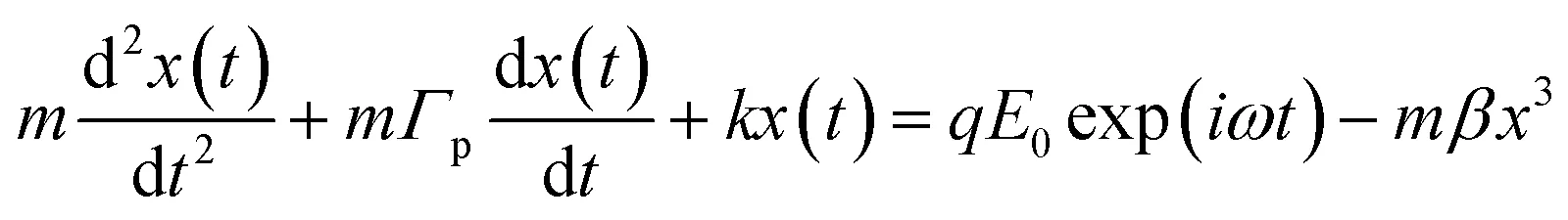


Following Landau's approach, this equation can be numerically solved, see the SI for some examples (Fig. S7).^4^ The resulting plasmonic absorption lineshape function has the following features: (i) it is notably asymmetrical; (ii) aside from the resonance condition at frequencies close to *ω*_p_, the nonlinearity causes additional resonances at rational multiples of *ω*_p_ (
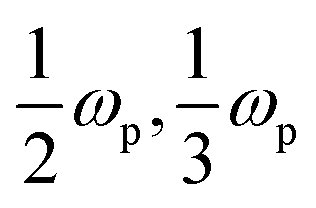
, *etc.*), that leads to the appearance of a series of “bumps” at the low-frequency side of the plasmonic absorption bands. These phenomena can result in asymmetry of plasmonic bands, as detailed in the experimental part of the UV-Vis plasmonic response.

To conclude this section, within the classical models of plasmonic absorption, there are two main factors generating the characteristic asymmetric plasmonic absorption bands in the currently studied NPs: (i) strong radiation damping and (ii) strong nonlinearity of the plasmonic oscillator.

#### 3.3.3. Quantum-mechanical models

Next, we show that asymmetry of UV-Vis plasmonic spectra can be fitted to a Fano lineshape function, naturally invoking the possibility to extend the classical modelling discussed above to a typical quantum phenomenon-the Fano-type resonance. Occurrence of Fano resonance involves interference of two different excitation pathways, one of which is continuous (continuous state-CS), while the other is discrete (discrete state-DS). In the present case, the plasmonic resonance (PR) plays the role of CS, while the most probable candidates for DS are electronic excitation of the NPs and/or excitation of a dark plasmon mode. In addition, by analogy to the results in ref. 46, vibrational excitations of surface adsorbed molecules/molecular ions have been also discussed to play the role of DSs.

Following the approach in ref. 45 and 46, we consider a discrete state |*d*〉 which is an eigenstate of an unperturbed Hamiltonian *Ĥ*_0_:

17 *Ĥ*_0_|*d*〉 = *E*_d_|*d*〉

which is coupled to a PR continuum state |*c*〉 through a coupling term *V̂*:18
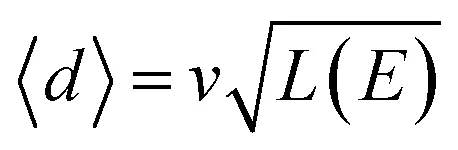
where *L*(*E*) denotes the plasmonic line-shape function with a maximum at *E*_p_ and width *Γ*_p_:19
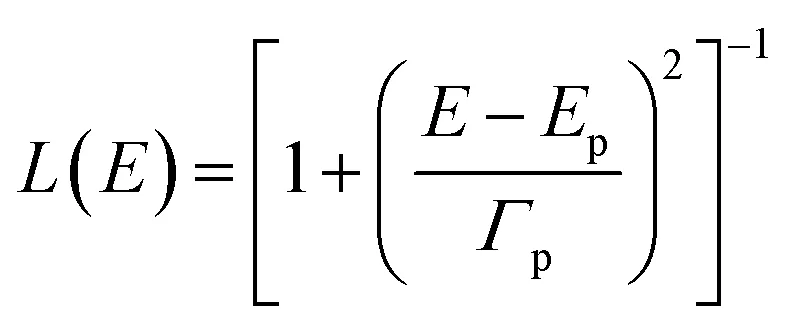


Introducing a new mixed quasi-continuum state |*Ψ*〉, which is an eigenstate of the perturbed Hamiltonian *Ĥ* = *Ĥ*_0_ + *V̂*, and considering an incident photon in a discrete state |*i*〉 which is coupled to |*d*〉 and |*c*〉 by an operator *Ŵ* with coupling factors *w* and *g* (〈*d*〉 = *w*, 
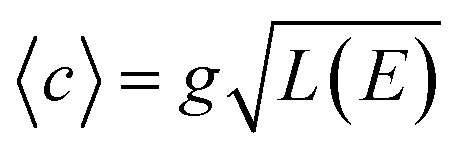
) it can be shown that the probability of the photon exciting the state |*Ψ*〉, which is normalized with respect to the corresponding probability of excitation of the continuum states in absence of DS, is given by Fano function of the form:^45,46^20
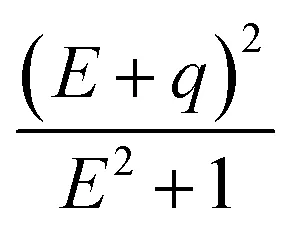


The parameters *E* and *q* (known as reduced energy and asymmetry factor, respectively) in eqn (20) are directly related to the plasmonic parameters, as following:21
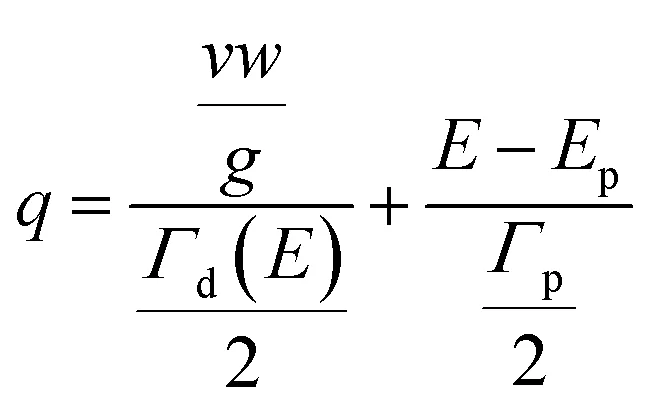
22
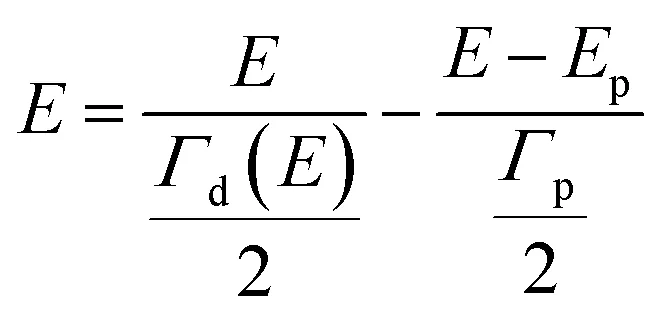
where

23 *Γ*_d_(*E*) = 2π*v*^2^*L*(*E*)

The last parameter is in close relation to the width of the discrete state. The above approach explicitly leads to a solution in which *q* and *E* are functions of all coupling factors as well as of the plasmon resonance energy and width. The outlined dependencies allow a description of possible scenarios at different ratios of coupling parameters and different relationships between plasmon resonance energy *E*_p_ and *E*_d_. The case when two resonances are characterized with same widths reduces to the so-called plasmon hybridization model.

Following the above discussion, the experimental UV-Vis optical absorption data were fitted with a generalized Fano-type function of the form (19), where the reduced energy *E* is defined as:24
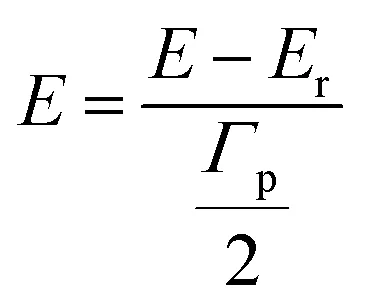


A typical fit is shown in Fig. 7. The results of the nonlinear least-square fitting of the optical absorption data are summarized in Table 3.

**Fig. 7:**
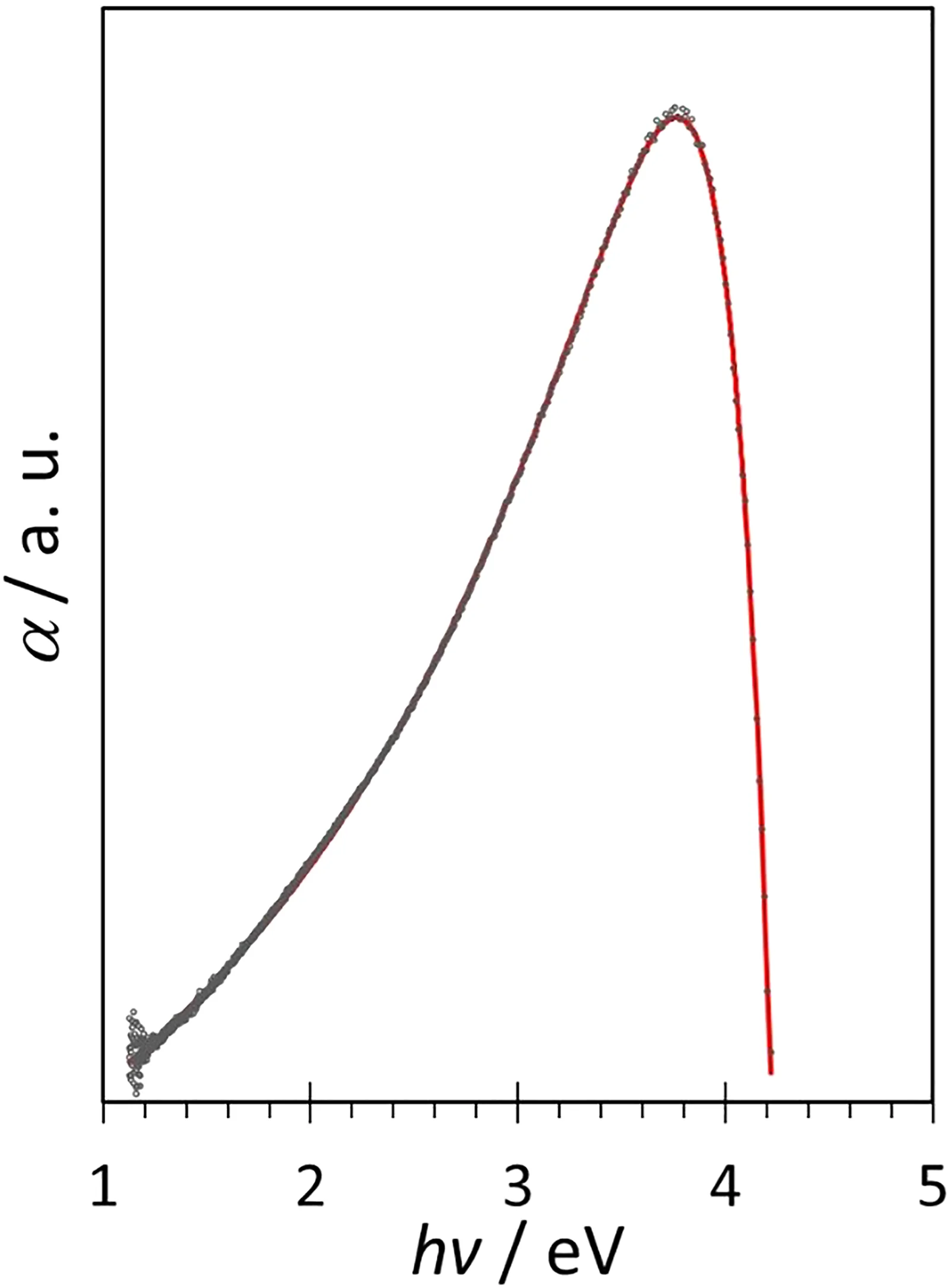
Plasmonic peak in the UV-VIS region of AgIn_5_S_8_:Zn-US together with a fit to a generalized Fano-type function (red line).

**Table 3:** The results of the nonlinear least-square fitting of the optical absorption data to the generalized Fano-type function of the form (7) for all NPs

	AgIn_5_S_8_-US	AgIn_5_S_8_	AgIn_5_S_8_:Zn	AgIn_5_S_8_:Zn-US
*Γ*_p_/eV	0.480 ± 0.006	0.839 ± 0.009	0.533 ± 0.005	0.527 ± 0.001
*E*_r_/eV	4.568 ± 0.007	4.461 ± 0.020	4.677 ± 0.007	4.360 ± 0.002
|*q*|/a.u.	0.598 ± 0.011	0.929 ± 0.027	0.645 ± 0.005	1.050 ± 0.004
*E*_max.-comp._	4.166 ± 0.007	4.010 ± 0.020	4.264 ± 0.007	4.109 ± 0.002

A small but notable shift in the PR energy *E*_r_ is observed upon doping. The plasmonic resonance width parameter *Γ*_p_ is significantly decreased on Zn doping, and use of US, reflecting the increased crystallinity observed through the decrease of the Urbach energy. The asymmetry parameter, which is proportional to the ratio of transition probabilities to the mixed state and to the continuum also shows a notable reduction for Zn doping and use of US, and is close to 1 for AgIn_5_S_8_:Zn-US. To account for the contribution of the electronic transitions to the overall spectral appearance above the optical band gap, we have also performed fitting of the optical spectra with a linear combination of Eliot-type function^50,51^ (which is an excellent approximation to the true spectral dependence of the absorption coefficient above the optical band gap) and Fano line shape functions. In each case (see the supplementary data), we have found that the contribution of the Eliot component was negligibly small and that the plasmonic absorption dominates the spectral appearance in this region. Therefore, the most plausible explanation of the asymmetric plasmonic absorption bands in the studied complex nanostructured systems is Fano-type resonance involving plasmonic states as CS and band-to-band electronic transitions as DS.

It is worth noting in this context that in the case of more complex structures, the apparent Fano-type asymmetry of the plasmonic bands can also be explained by generalization of the concept of plasmon hybridization originally developed in ref. 43 and 44. This approach can be generalized and applied to more complex multifeatured plasmon response such as concentric nanoshells, nanoparticle aggregates *etc.*

While the present study focuses on the manifestations of plasmon-mediated asymmetry in absorption spectra of the studied nanocrystals, the underlying electrodynamic mechanisms naturally extend to excited-state phenomena. For example, electromagnetic reciprocity relates far-field absorption to far-field emission *via* the dyadic Green's function. The established foundational mechanisms of plasmon generation and its manifestation in absorption cross-sections are equally fundamental to emission, as a consequence of underlying interference between electric and magnetic multipoles. Asymmetric emission can be in principle expected from the same symmetry-broken mode structure as it is governed by the radiative local density of optical states (LDOS). An in-depth study of the optical emission phenomena in the series of studied system is in progress.

## 4. Conclusion

In conclusion, we have reported widely tunable plasmonic resonance properties in ternary Ag–In–S and quaternary Ag–In–S–Zn semiconductor nanostructures. We demonstrated that these materials exhibit localized surface plasmon resonance (LSPR) in the NIR and, significantly, interband plasmon in the UV spectral regions (300–350 nm), extending semiconductor plasmonic to shorter wavelengths.

We have shown that these plasmonic properties can be precisely tailored by controlling the free charge carrier concentration, and overall, their electronic properties such as band gaps and Urbach energies. This control is achieved *via* a bottom-up colloidal approach utilizing two distinct strategies: extrinsic Zn-doping and the application of an ultrasound field during synthesis to control crystallinity and structural disorder. A key finding is the UV energy of the interband plasmons in all NPs and their different asymmetric nature in contrast to the symmetric NIR LSPR bands. The UV plasmonic peaks are excellently modeled with a Fano-like band shape function, indicating their quantum mechanical origin.

This asymmetry can be understood using two frameworks. Quantum-mechanically, it is attributed to a Fano-like resonance between a continuous plasmon state and a discrete electronic excitation *i.e.* exciton or dark plasmon mode. Classically, it corresponds to a driven-damped oscillator with a nonlinear restoring force or radiation damping term.

By interpolating the experimental LSPR bands with a generalized Fano line shape function, all relevant parameters characterizing the resonance such as plasmon damping, resonance energy and asymmetry factor were obtained. The demonstrated plasmon-exciton coupling in these Ag–In–S-based NPs, tunable *via* both external doping and sonochemical processing, offers a new and robust platform for applications requiring precise control of plasmon absorption across a wide spectral region.

## Author contributions

Biljana Pejova: conceptualization, investigation, writing – original draft, writing – review & editing, funding acquisition, methodology, visualization, project administration, data curation, supervision, resources. Julio A. do Nascimento: investigation, writing – review & editing, data curation. Rola Moussa: investigation, writing – review & editing, data curation. Fayzah Talbi: investigation, writing – review & editing, data curation. Adam Kerrigan: investigation, writing – review & editing, methodology, data curation, supervision, resources. Leonardo Lari: investigation, writing – review & editing, methodology, data curation, supervision, resources. Richard Douthwaite: investigation, writing – review & editing, methodology, data curation, supervision, resources. Ljupcho Pejov: conceptualization, investigation, writing – review & editing, funding acquisition, methodology, visualization, project administration, data curation, supervision, resources, software. Vlado Lazarov: conceptualization, investigation, writing – original draft, writing – review & editing, funding acquisition, methodology, visualization, project administration, data curation, supervision, resources.

## Conflicts of interest

There are no conflicts to declare.

## Supplementary Material

TC-OLF-D6TC02675C-s001

## Data Availability

Supplementary information (SI) is available. See DOI: https://doi.org/10.1039/d6tc02675c. The datasets generated and analyzed during the current study are available from the authors on reasonable request.
