# Supplementary material for: Extending semiconductor plasmonics to the UV: Fano resonances and disorder engineering in Ag–In–S nanoparticles
Source: J Mater Chem C Mater. 2026 Oct 26. Online ahead of print. doi: 10.1039/d6tc02675c (PMC13728871; doi:10.1039/d6tc02675c)
Supplement: Supplementary file 1 [file TC-OLF-D6TC02675C-s001.pdf]

Supplementary Information (SI) for Journal of Materials Chemistry C.
This journal is © The Royal Society of Chemistry

Supporting Information

Extending Semiconductor Plasmonics to the UV: Fano Resonances and Disorder Engineering in Ag-In-S Nanoparticles

Biljana Pejova[1*], Julio A. do Nascimento[2], Rola Moussa[2], Fayzah Talbi[3], Adam Kerrigan[2,4], Leonardo Lari[2,4,5], Richard Douthwaite[6], Ljupcho Pejov[1], Vlado K. Lazarov[2,4*]

[1]Institute of Chemistry, Faculty of Natural Sciences and Mathematics, SS. Cyril and Methodius University, POB 162, 1000 Skopje, North Macedonia

[2]School of Physics Engineering and Technology, University of York, York, YO10 5DD, UK

[3]Department of Physical Sciences, Faculty of Science, University of Jeddah, P.O. Box 13151, Jeddah, 21493, Saudi Arabia

[4]The York-JEOL Nanocentre, University of York, York, YO10 5DD, UK

[5]Area Science Park, Padriciano 99, 34149, Trieste, Italy

[6]Department of Chemistry, University of York, York, YO10 5DD, UK

E-mail: biljana@pmf.ukim.mk

E-mail: vlado.lazarov@york.ac.uk

SI Table 1. The relevant parameters of the plasmon bands ($\square_p$ and $\square_p$) obtained by the nonlinear least-square fitting of the optical absorption data in the NIR spectral region to the Cauchy-Lorentzian-type function of the form (4), together with the computed values of the dephasing times – see text for details.

	$AgIn_5S_8$-US	$AgIn_5S_8$	$AgIn_5S_8$:Zn	$AgIn_5S_8$:Zn-US
$\square_p$ / eV<br>($\square_p$ / eV; $\square$ / fs)	0.5542<br>(0.0172; 76.4)	0.5518<br>(0.0198; 66.4)	0.5545<br>(0.0268; 49.2)	0.5530<br>(0.0279; 47.1)
		0.5608<br>(0.0149; 88.6)		
	0.5648<br>(0.0248; 53.2)	0.5675<br>(0.0109; 120.7)	0.5671<br>(0.0154; 85.6)	0.5668<br>(0.0159; 82.8)
	0.6198<br>(0.0358; 36.8)	0.6181<br>(0.0325; 40.5)	0.6164<br>(0.0310; 42.5)	0.6198<br>(0.0386; 34.1)
	0.6349<br>(0.0220; 59.9)	0.6350<br>(0.0263; 50.1)	0.6315<br>(0.0263; 50.1)	0.6359<br>(0.0231; 57.0)
	0.6434<br>(0.0155; 85.1)	0.6429<br>(0.0114; 115.8)	0.6429<br>(0.0157; 83.7)	0.6446<br>(0.0153; 86.2)
		0.6715<br>(0.1173; 11.2)		
	0.6868<br>(0.0545; 24.1)		0.6853<br>(0.0598; 22.0)	0.6849<br>(0.0578; 22.8)
		0.7301<br>(0.0035; 371.2)		
	0.7382<br>(0.0298; 44.2)	0.7358<br>(0.0098; 134.2)	0.7368<br>(0.0102; 129.6)	0.7356<br>(0.0108; 121.9)
		0.7454<br>(0.0049; 266.8)	0.7481<br>(0.0229; 57.5)	0.7465<br>(0.0077; 172.1)
	0.7508<br>(0.0298; 44.2)	0.7493<br>(0.0130; 101.6)	0.7570<br>(0.0035; 371.2)	0.7502<br>(0.0217; 60.6)
		0.7620<br>(0.0185; 71.3)	0.7613<br>(0.0235; 56.1)	
	0.8402<br>(0.0644; 20.5)		0.8320<br>(0.0756; 17.4)	0.8397<br>(0.0585; 22.5)
	0.8665<br>(0.0292; 45.1)		0.8681<br>(0.0288; 45.7)	0.8691<br>(0.0277; 47.6)
		0.8709<br>(0.0280; 47.1)		

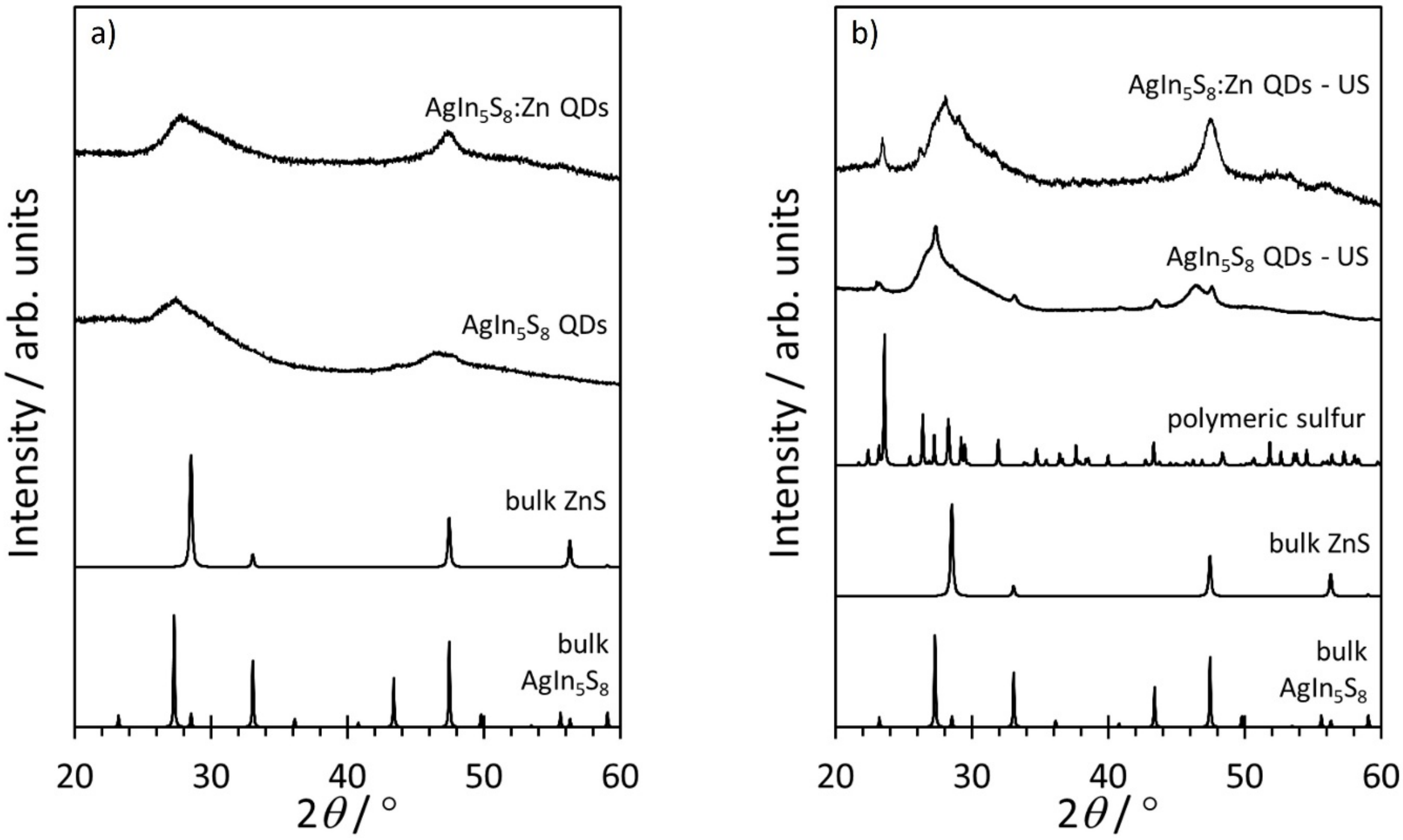

SI-Figure 1. XRD patterns of: a) undoped ($AgIn_5S_8$) and doped ($AgIn_5S_8$:Zn) NPs prepared in the absence of ultrasound and b) undoped and doped QDs prepared under US ($AgIn_5S_8$ NPs-US and $AgIn_5S_8$:Zn NPs-US) compared with XRD patterns of bulk $AgIn_5S_8$, ZnS and polymeric sulfur.

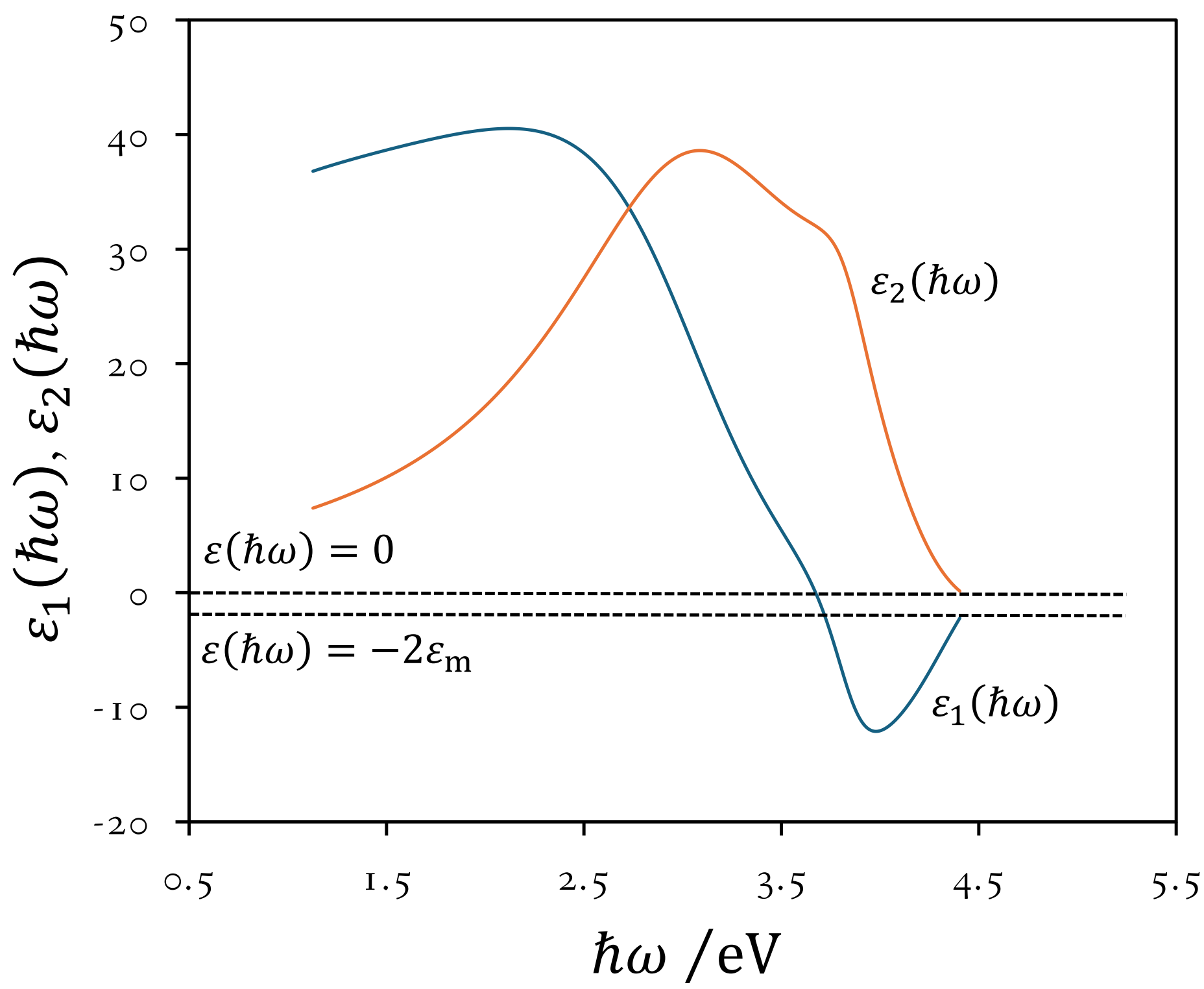

SI-Figure 2. Real and imaginary components of the complex dielectric function for undoped $AgIn_5S_8$ NCs obtained from optical spectroscopy data (ε_m = 1, no surface attached ligands).

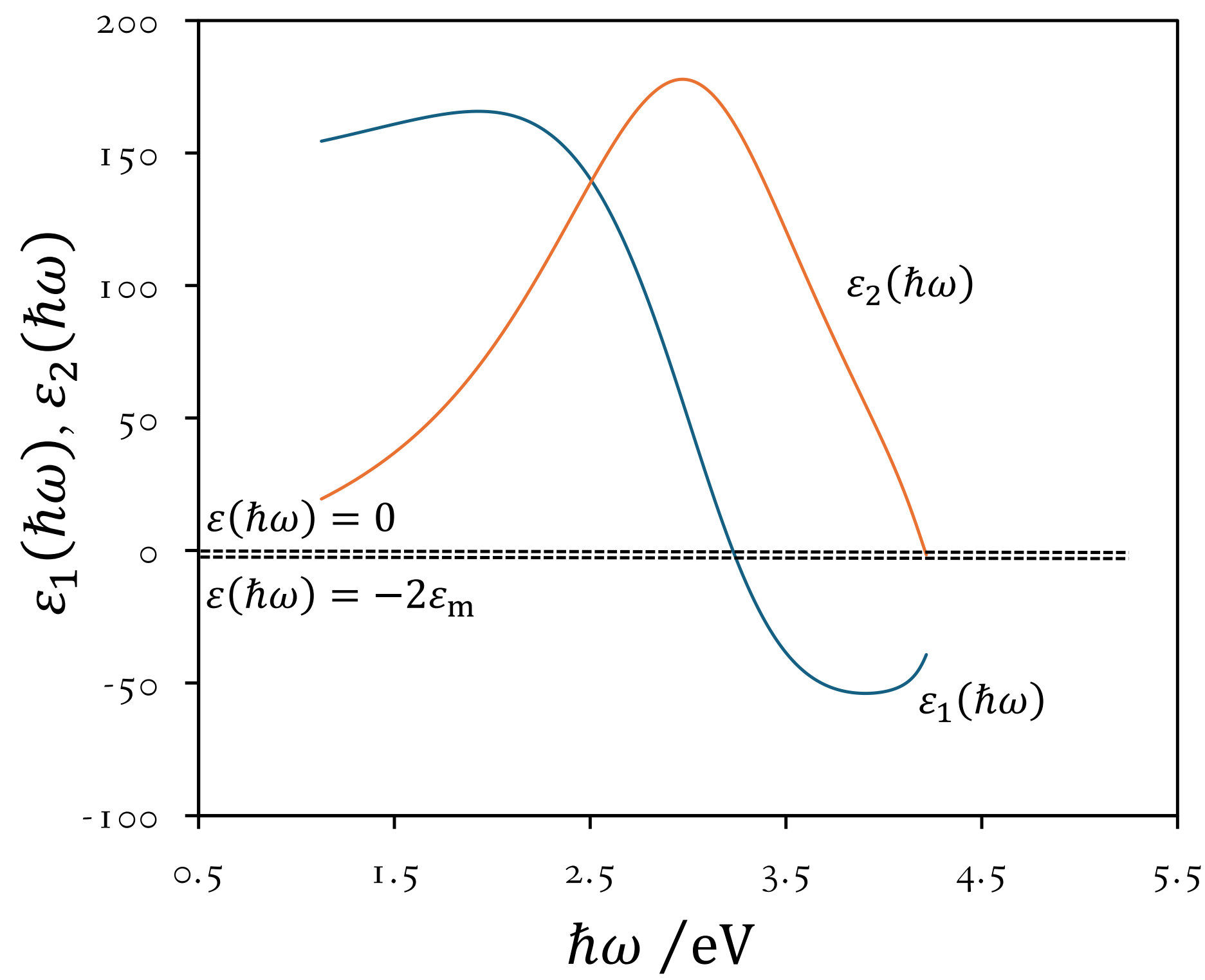

SI-Figure 3. Real and imaginary components of the complex dielectric function for undoped $AgIn_5S_8$ NCs obtained from optical spectroscopy data (ε_m = 1, no surface attached ligands).

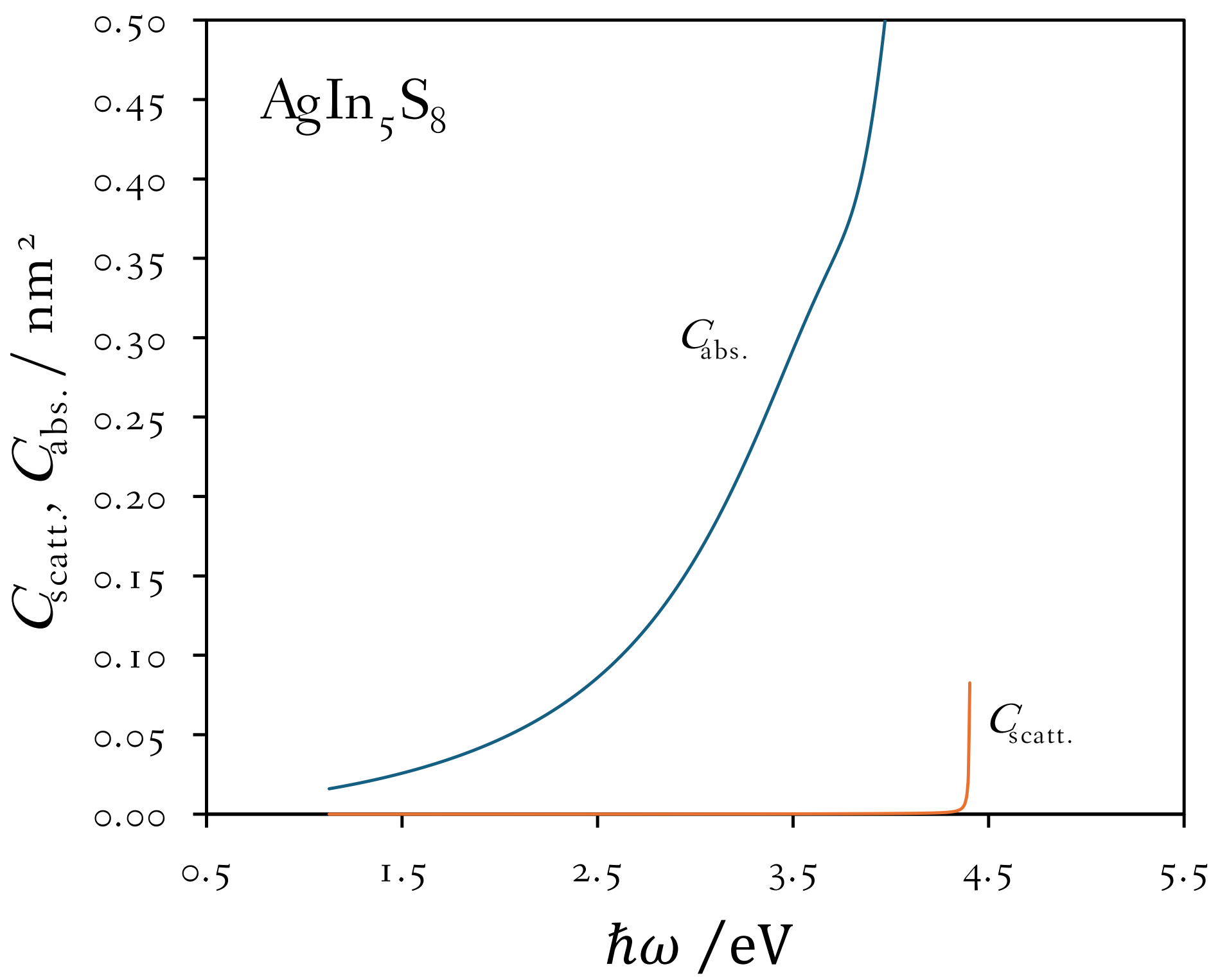

SI-Figure 4. Relative contributions of scattering and absorption to the optical response of undoped $AgIn_5S_8$ and $AgIn_5S_8$:Zn NPs evaluated using a dielectric function – based electromagnetic model.

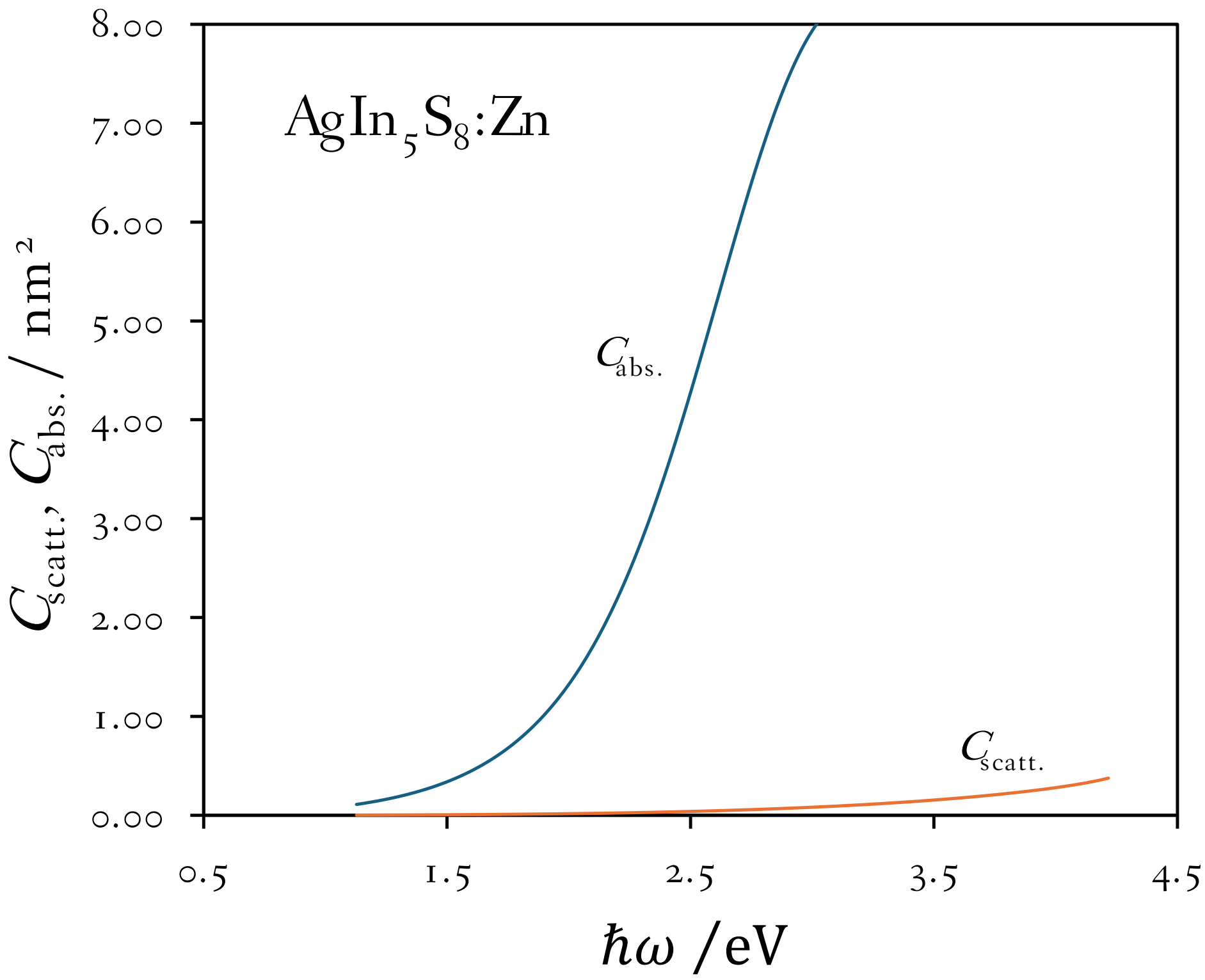

SI-Figure 5. Relative contributions of scattering and absorption to the optical response of undoped $AgIn_5S_8$ and $AgIn_5S_8$:Zn NPs evaluated using a dielectric function – based electromagnetic model.

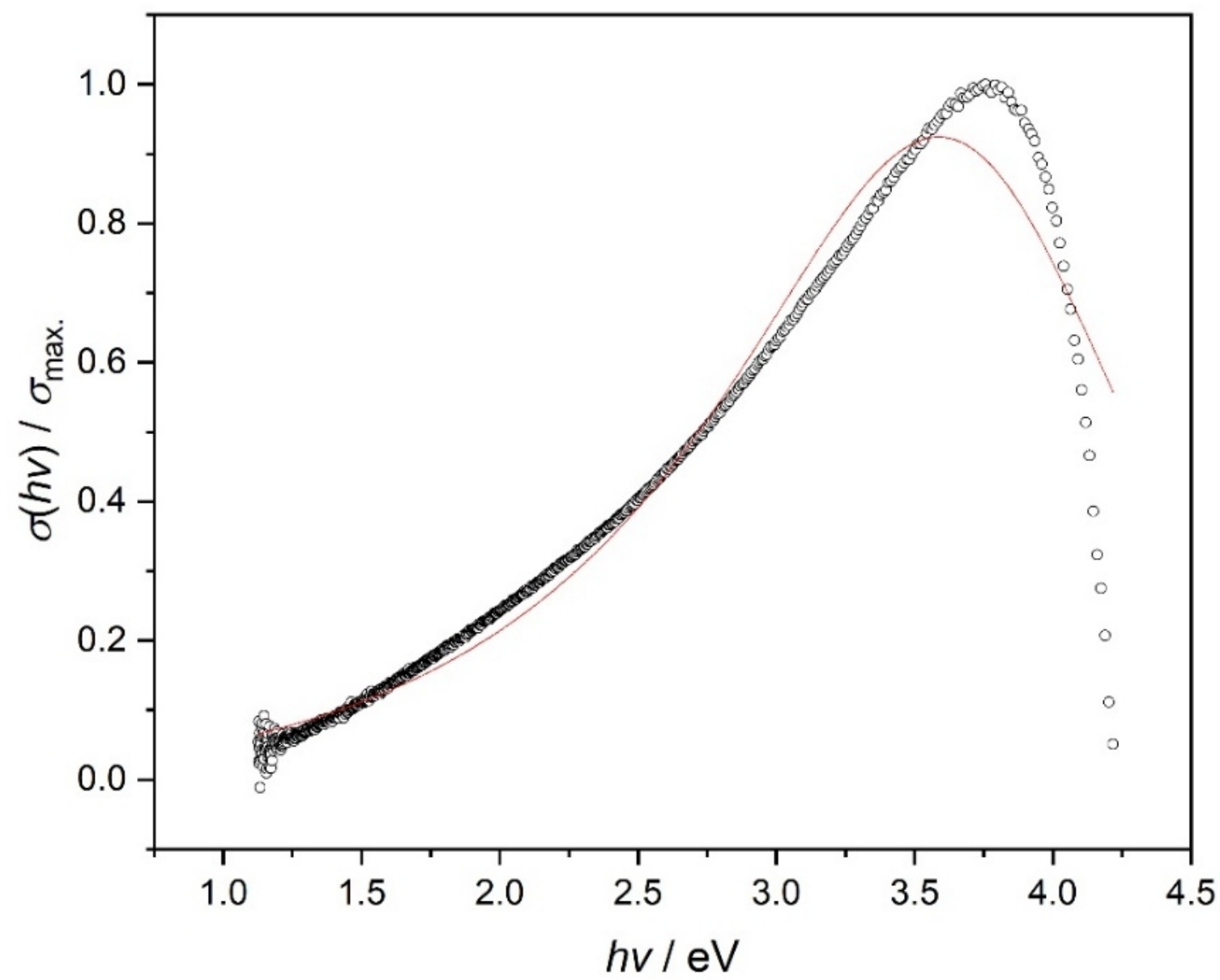

SI-Figure 6. Plasmonic peak in the UV-VIS region of $AgIn_5S_8$-US together with a fit to a function of the form (9) (red line), arising from the classical oscillator model involving radiation damping.

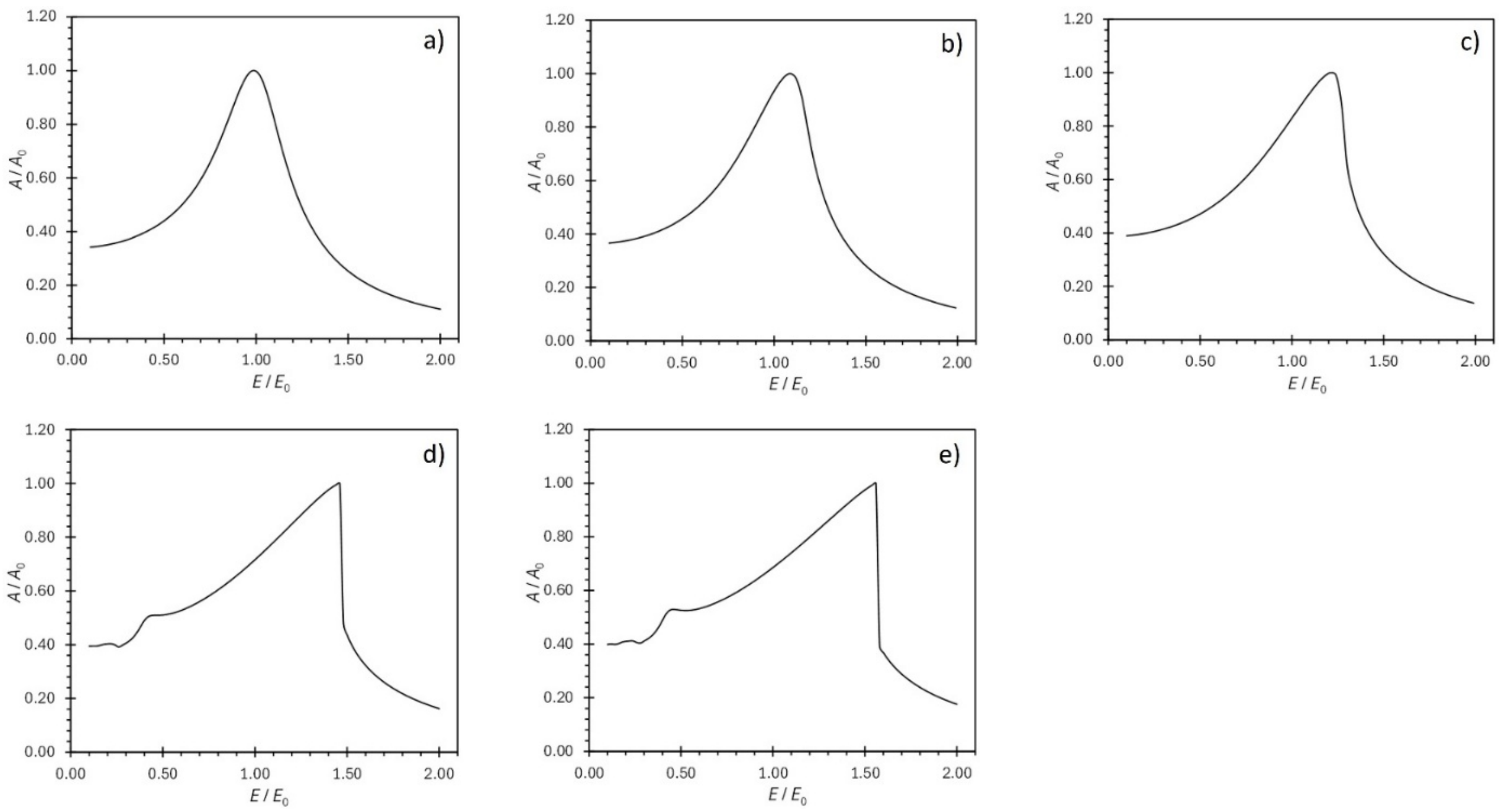

SI-Figure 7. Normalized lineshape functions arising from Landau's solution of the equation of motion for non-linear driven-damped oscillator: $-qE_0$ = a) 0.1, b) 0.3, c) 0.5, d) 0.9, e) 1.1.

Calculation of the absorption and scattering cross-sections

For spherical nanoparticles, much smaller than the wavelength of the incident radiation, the quasistatic approximation can be used. Effectively, the NP is treated as an induced electric dipole in the incident field. The complex polarizability is given by:[23]

$$\alpha_v(\hbar\omega) = 4\pi a^3 \frac{\varepsilon(\hbar\omega) - \varepsilon_m}{\varepsilon(\hbar\omega) + 2\varepsilon_m} \quad \text{(SI-1)}$$

where a is the NP radius while ε_m is the relative permittivity of the surrounding medium. The complex dielectric function has the form:

$$\varepsilon(\hbar\omega) = \varepsilon_1(\hbar\omega) + i\varepsilon_2(\hbar\omega) \quad \text{(SI-2)}$$

The quasistatic dipole expression for the absorption cross-section is:

$$C_{abs.} = k_m \cdot \mathrm{Im}\left[\alpha_v(\hbar\omega)\right] \quad \text{(SI-3)}$$

where:

$$k_m = \frac{2\pi n_m}{\lambda_o} \quad \text{(SI-4)}$$

The scattering cross-section for an electric dipole in a nonabsorbing surrounding medium is given by:

$$C_{scatt.} = \frac{k_m^4}{6\pi}\left|\alpha_v(\hbar\omega)\right|^2 \quad \text{(SI-5)}$$

where:

$$\left|\alpha_v(\hbar\omega)\right|^2 = \left\{\mathrm{Re}\left[\alpha_v(\hbar\omega)\right]\right\}^2 + \left\{\mathrm{Im}\left[\alpha_v(\hbar\omega)\right]\right\}^2 \quad \text{(SI-6)}$$
